# Tailoring e_g_ Orbital Occupancy of Fe in Ni-Doped Na_4.3_Fe_3_(PO_4_)_2_P_2_O_7_ Cathode for High-Performance Sodium-Ion Batteries

**DOI:** 10.1007/s40820-026-02073-3

**Published:** 2026-02-05

**Authors:** Xiaoxue Wang, Yuhui Xu, Jianhua Zhang, Yukun Xi, Ningjing Hou, Yixuan Chen, Dongzhu Liu, Zihao Yang, Haocheng Wen, Jia Kang, Xiaoli Yang, Xuexia Song, Jingjing Wang, Wenbin Li, Jiujun Zhang, Kun Zhang, Xifei Li

**Affiliations:** 1 Institute of Advanced Electrochemical Energy & School of Materials Science and Engineering, Xi’an University of Technology, Xi’an, 710048 Shaanxi People’s Republic of China; 2 Shaanxi Engineering Research Center of Key Materials for Lithium/Sodium-Ion Batteries, Xi’an University of Technology, Xi’an, 710048 Shaanxi People’s Republic of China; 3 Institute of New Energy Materials and Engineering, College of Materials Science and Engineering, Fujian Engineering Research Center of High Energy Batteries and New Energy Equipment & Systems, Fuzhou University, Fuzhou, 350108 Fujian People’s Republic of China; 4 GEM Co., Ltd., Shenzhen, 518101 Guangdong People’s Republic of China; 5 Hubei Provincial Key Laboratory of High-Value Utilization of Retired Power Batteries, Jingmen, 448000 Hubei People’s Republic of China

**Keywords:** Na_4.3_Fe_3_(PO_4_)_2_P_2_O_7_, Electronic coupling, e_g_ orbital occupancy, Descriptor, Sodium-ion batteries

## Abstract

Na_4_Fe_3_(PO_4_)_2_P_2_O_7_ (NFPP) is regarded as a prospective cathode for sodium-ion batteries (SIBs) because of its high structural stability and cost-effectiveness. However, its practical application is hindered by intrinsically low electronic conductivity. Herein, an unconventional electron transfer mechanism from Ni^2+^ to Fe^3+^ ions is unveiled in Ni-doped Na_4.3_Fe_3_(PO_4_)_2_P_2_O_7_ (NFPP-Ni) cathode, which facilitates electronic coupling within the Fe−O−Ni coordination unit and thereby effectively boosts electron transport. Moreover, the redox kinetics and reversibility of NFPP materials are predominantly governed by the degree of Fe−O covalency. The intermediate e_g_ occupancy of Fe^2+^, modulated by the presence of Ni^2+^, optimizes the overlap between Fe d and O p orbitals. The adjustment of Ni dopant strikes a balance between accelerating Na^+^ diffusion kinetics and mitigating lattice strain during cycling. As a result, the NFPP-Ni electrode displays impressive rate capacity (121.0 mAh g^−1^ at 0.1C / 80.9 mAh g^−1^ at 10C) and stable cyclability (89.1% capacity retention after 1000 cycles). More importantly, the relationship between Fe e_g_ orbital occupancy and Fe−O covalency in NFPP as modulated by various transition metal cations (Ni^2+^, Mn^2+^, Zn^2+^, Co^2+^ and Cu^2+^) with different electron configurations are systematically elucidated, thereby providing insights for the commercial development of sodium-ion batteries (SIBs). Tuning the e_g_ orbital occupancy of Fe in Na_4.3_Fe_3_(PO_4_)_2_P_2_O_7_ cathode can effectively optimize the spatial overlap between Fe d and O p orbitals with excellent rate capability for sodium-ion batteries. The e_g_ could be a significant descriptor for Fe−O covalency that describes a volcano curve as a function of e_g_.

**Supplementary Information:**

The online version contains supplementary material available at 10.1007/s40820-026-02073-3.

## Highlights

- The Na_4.3_Fe_3_(PO_4_)_2_P_2_O_7_-M cathode constructed by various transition metal cations (M = Ni^2+^, Mn^2+^, Zn^2+^, Co^2+^ and Cu^2+^) with different electron configurations for sodium-ion batteries.
- The Na_4.3_Fe_3_(PO_4_)_2_P_2_O_7_-Ni cathode exhibits superior electronic conductivity, high-rate performance and stable cyclability.
- A quantitative relationship between the eg occupancy of Fe and the electrochemical activity of The Na_4.3_Fe_3_(PO_4_)_2_P_2_O_7_-M is proposed, serving as an activity descriptor.

**Supplementary Information:**

The online version contains supplementary material available at 10.1007/s40820-026-02073-3.

## Graphical abstract

**Figure:**
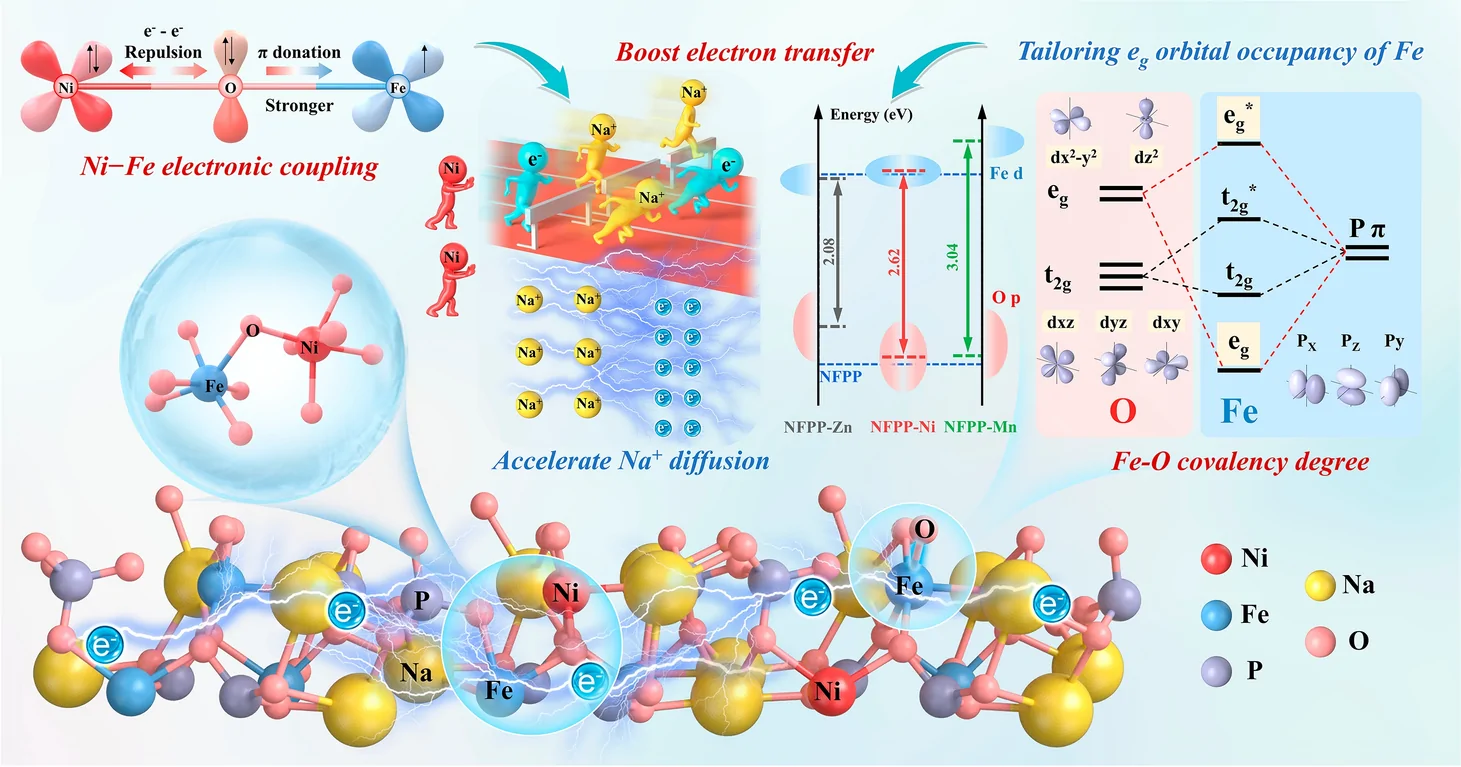

## Introduction

As the explosive expansion of electronic devices and energy storage demands, sodium-ion batteries (SIBs) have emerged as a potential alternative to lithium-ion batteries, attributed to the wide distribution and high abundance of Na as well as its chemically similar properties to Li [1–3]. Considering the decisive effect of cathode materials in deciding the electrochemical property of batteries, multiple high-specific-energy cathode candidates have been developed, mainly including oxides, polyanionic compounds, and Prussian blue [4–6]. Among these, Na_4_Fe_3_(PO_4_)_2_P_2_O_7_ (NFPP), a representative polyanionic cathode with a sodium superionic conductor (NASICON) structure, exhibits excellent structural stability and rapid ion diffusion kinetic due to its open three-dimensional framework [7, 8]. In addition, the Fe^2+^/Fe^3+^ redox center in NFPP is non-toxic, earth-abundant, and cost-effective, further enhancing its appeal as a cathode material. NFPP has thus attracted considerable scholarly attention in recent years.

It is well established that electron transport in polyanionic materials, governed by the “M−O−P−O−M” pathway (where M denotes the redox center), is inherently sluggish due to their distinctive crystal structures, leading to poor conductivity [9]. The inherently low electronic conductivity of NFPP cathodes greatly restricts their practical electrochemical performance. In response, various NFPP/C composites were developed to enhance the conductivity of the material [10–12]. However, such approaches often overlook the improvement of the intrinsic conductivity and reaction kinetics of NFPP itself. Element doping/substitution has been proven as an effective approach for fundamentally increasing the internal electronic conductivity of materials [13, 14], because the electrochemical property of electrode is primarily depended on the electronic structure of their electrochemically active centers. Specifically, the co-doping of Mn^2+^ and F^−^ can be used to adjust the electronic spin state of the electrochemical active center Fe^2+^, thereby promoting electron transport [15]. An orbital-delocalization-assisted valence modulated strategy has also been proposed to enhance the electronic conductivity of NFPP materials. This is because the partially filled 3d orbitals in Mo^6+^ introduce additional electronic states into the conduction band [16]. Additionally, the electroactive coefficient (η) is introduced to evaluate the relative contribution of electrons and ions to the transport capacity. The V-doping strategy triggers a high-spin-low-spin transition of Fe^2+^, enhancing multi-electron transfer and increasing η to 0.85 [17]. Despite this, there are few studies that explore how different electronic configurations of doped ions regulate the lattice structure and electronic distribution of polyanionic compounds to facilitate electron transport and reversible ion diffusion. Recently, the reductive coupling mechanism (RCM) between transition metal ions (TM^n+^) and O induced by elemental doping has been reported in sodium-ion oxide cathodes [18]. In the field of electrocatalysis, differences in e_g_ orbital filling have been shown to affect the binding strength of catalysts to oxygen intermediates, serving as a crucial descriptor of catalytic activity [19, 20]. Therefore, it is significant to investigate how doping TM^n+^ can favorably modulate the orbital electronic distribution of active center (Fe) in NFPP materials.

**Fig. 1:**
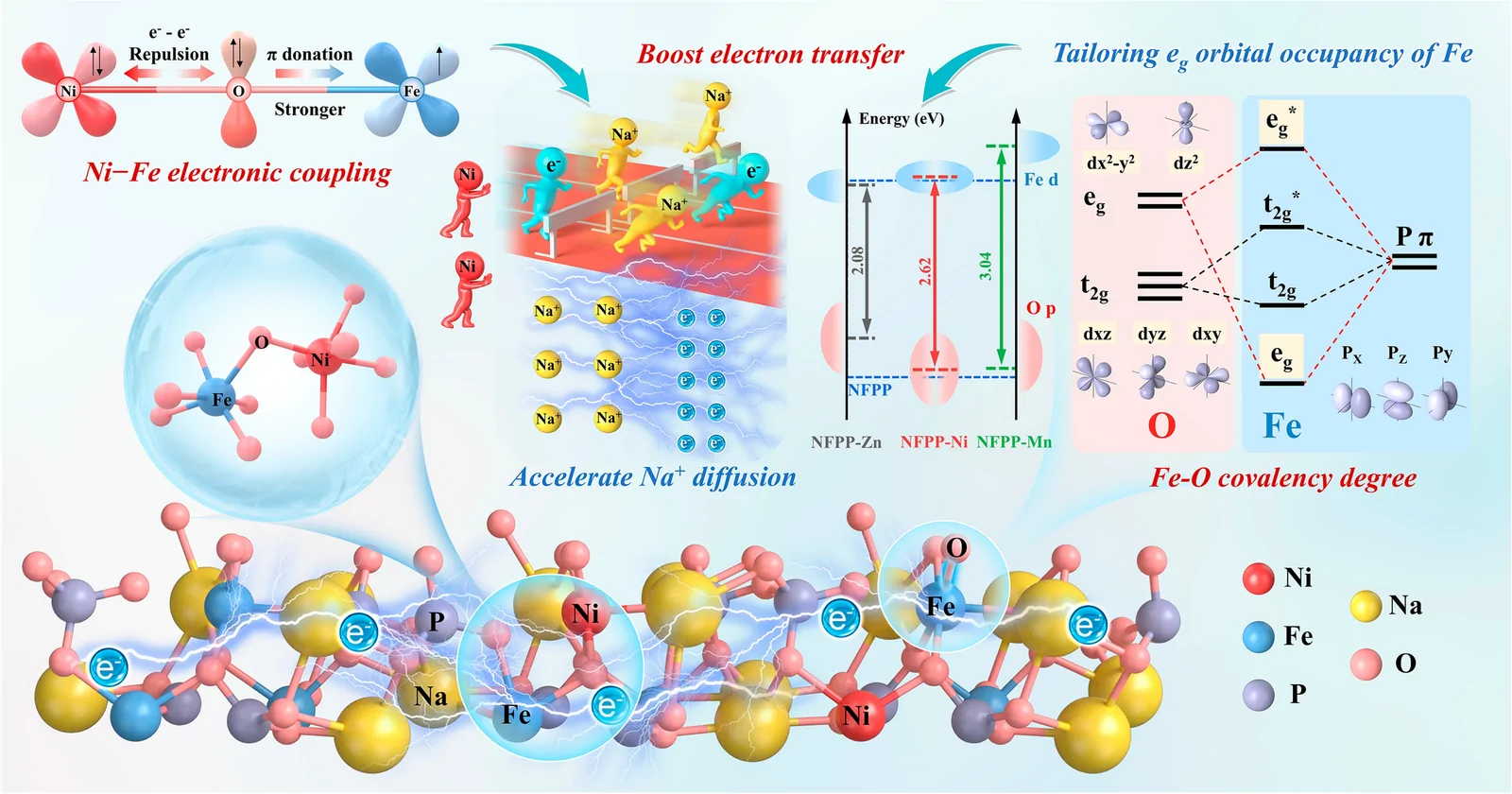
Schematic illustration of Ni−Fe electronic coupling and changes in e_g_ orbital occupancy in the NFPP cathode following Ni doping

In this work, a strategy for regulating e_g_ orbital occupancy via electronic coupling is proposed to modulate the electron transport in Na-enriched Na_4.3_Fe_3_(PO_4_)_2_P_2_O_7_ cathode by employing dopant ions with different electronic structures (i.e., Ni^2+^, Mn^2+^, Zn^3+^, Co^2+^ and Cu^2+^). Notably, the optimization of Fe−O covalent bonding is triggered by changes in e_g_ orbital occupancy resulting from Ni−Fe electronic coupling, thereby synergistically enhancing electron transport and accelerating Na^+^ insertion/extraction kinetics (Fig. 1). The spatial overlap of the Fe d orbitals with the O p orbitals reaches the optimal state in the presence of Ni^2+^, achieving superior specific capacity and satisfied cycling stability (89.1% of capacity retention after 1000 cycles). Overall, this work offers new perspectives for designing reliable and advanced cathode for SIBs.

## Experimental Section

### Materials

Both the samples were prepared via typical sol–gel methods. All the chemicals are directly used in this paper after purchase without further purification. Citric acid (C_6_H_8_O_7_, ≥ 99.5%), C_2_H_3_NaO_2_ (≥ 98.5%), NH_4_H_2_PO_4_ (≥ 99.0%), ethylene glycol (C_2_H_6_O_2_, ≥ 99.5%), C_4_H_6_NiO_4_·4H_2_O (≥ 98.0%), C_4_H_6_MnO_4_ (≥ 98.0%), C_4_H_6_ZnO_4_·2H_2_O (≥ 99.0%) were purchased from Sinopharm Group Chemical Reagent Co., Ltd., China. C_4_H_6_FeO_4_ (≥ 90.0%) and N-methylpyrrolidone (NMP, 99.9%) were purchased from Aladdin Reagent (Shanghai) Co., Ltd., China.

### Synthesis of Na_4.3_Fe_3_(PO_4_)_2_(P_2_O_7_)-M (M = Ni, Mn, Co, Cu or Zn) samples

In a typical process, the stoichiometric Fe, Ni/Mn/Zn, Na, P sources and citric acid were added into the deionized (DI) water in a molar ratio of 1.95: 0.067: 2.89: 2.69: 2.52. The mixed solution was heated and stirred in an oil at 90 °C for 0.5 h to fully react and then heated and stirred at 120 °C for 1 h to completely remove the solvent. The precursor was ground into the fine powder and then annealed at 550 °C for 8 h under Ar/H_2_ gas atmosphere to get the final products. The original Na-enriched Na_4.3_Fe_3_(PO_4_)_2_(P_2_O_7_) and Ni^2+^-, or Mn^2+^-, or Co^2+^-, or Cu^2+^-, or Zn^2+^-doped Na_4.3_Fe_3_(PO_4_)_2_(P_2_O_7_) were abbreviated as NFPP, NFPP-Ni, NFPP-Mn, NFPP-Co, NFPP-Cu, and NFPP-Zn, respectively.

### Characterizations

The surface morphology, elemental distribution, and microstructure of the samples were measured by scanning electron microscopy (SEM, ZEISS Gemini 300), energy-dispersive spectroscopy (EDS) elemental maps, and transmission electron microscopy (TEM, FEI Talos F200X). The crystallographic information was characterized by X-ray diffractometer (XRD, SmartLab) with a monochromated CuKa X-ray source. The corresponding schematic illustrations were presented by VESTA software based on the refined results. The contents of Fe, Ni, and Mn in different samples were determined by inductively coupled plasma (ICP-OES, Agilent 5110). The thermal stability measurements were taken at air atmosphere with a heating rate of 10 °C min^−1^ from room temperature to 800 °C using a simultaneous thermal analyzer (HITACHI STA200). The electronic conductivities were carried out using four-terminal method ST2722-SD with ST2643 high-impedance test. The chemical composition and valence of the samples were investigated using X-ray photoelectron spectroscopy (XPS, Thermo ESCALAB 250XI). Raman spectra were measured on a confocal laser microscope Raman spectrometer (HORIBA Scientific LabRAM HR Evolution) with an excitation wavelength of 532 nm to monitor the molecular state and structure. Diffuse reflection spectra of UV–Vis (UV–Vis DRS) were characterized using a UV-3600 Plus spectrometer (UV-3600IPLUS, 220 C (A12615900129)). The X-ray absorption spectra (XAS) including X-ray absorption near-edge structure (XANES) and extended X-ray absorption fine structure (EXAFS) of the sample at Fe/Ni K-edge were collected at the Beamline of TLS07A1 in National Synchrotron Radiation Research Center (NSRRC), Taiwan. The in situ XRD experiment was performed on a PANalytical (Empyrean) diffractometer equipped with a CuKα radiation. The details of electrochemical measurements and theoretical calculations can be found in the experimental section of the Supplementary Information.

## Results and Discussion

### Material Characterization

**Fig. 2:**
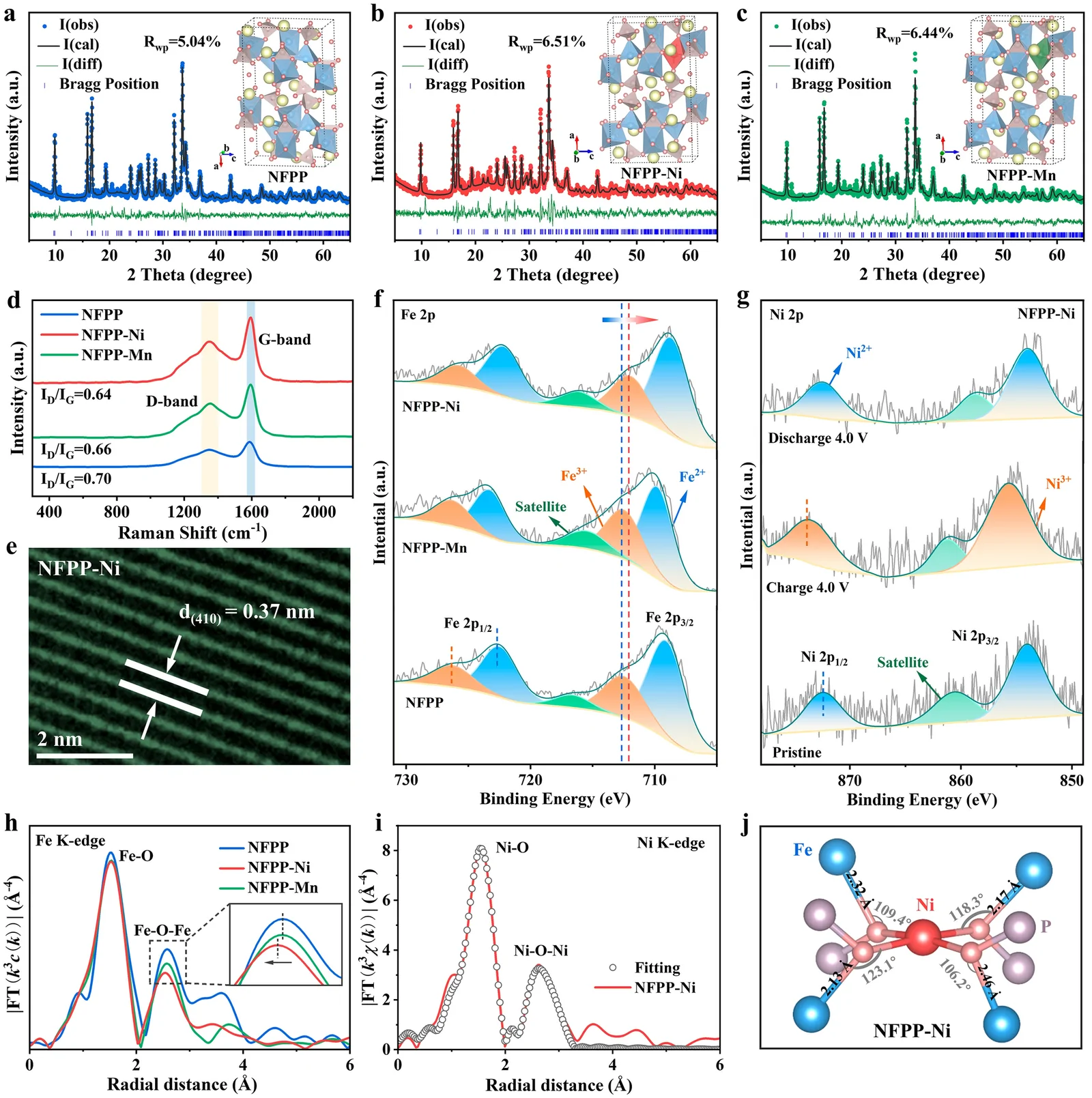
Rietveld refinements of **a** NFPP, **b** NFPP-Ni, and **c** NFPP-Mn. The insets are corresponding crystal structures based on the refinement results (blue, red, and green octahedra correspond to FeO_6_, NiO_6_, and MnO_6_, respectively; purple tetrahedra, yellow spheres, and red spheres represent PO_4_, Na, and O atoms, respectively). **d** Raman spectra. **e** HRTEM images. **f** Fe 2*p* and **g** Ni 2*p* XPS spectra for NFPP-Ni at different electrochemical states. **h** Fe K-edge and **i** Ni K-edge FT-EXAFS. **j** Calculated atomic structure variation of NFPP-Ni

Both M^2+^-doped Na-enriched Na_4.3_Fe_3_(PO_4_)_2_P_2_O_7_ (where M represents Ni, Mn, Co, Cu, and Zn, denoted as NFPP-Ni, NFPP-Mn, NFPP-Co, NFPP-Cu, and NFPP-Zn, respectively) and pure Na_4.3_Fe_3_(PO_4_)_2_P_2_O_7_ (NFPP) are obtained by the sol–gel method (Fig. S1). The XRD spectra of all samples (Fig. S2) show good agreement with the standard PDF card (PDF#: 89–0579). This suggests that the introduction of a small amount of dopant has a negligible impact on the overall crystal structure. Figure 2a-c presents the satisfactory Rietveld refinement results for the XRD pattern of these three samples. All samples exhibit well-weighted profile R factors (R_wp_ = 5.04% for NFPP, 6.51% for NFPP-Ni, and 6.44% for NFPP-Mn), which are consistent with previously reported values for NFPP (orthorhombic, spatial group: Pn2_1_a) [21]. Further analysis confirms that the dopants (Ni and Mn) are successfully incorporated at the Fe sites within the structure. The doping content of Ni/Mn is measured via ICP-OES (Table S1). Detailed crystallographic parameters are presented in Tables S2–S5. The representative NASICON open framework of NFPP, NFPP-Ni, and NFPP-Mn is illustrated in Fig. 2a-c. In this structure, layers of [Fe_3_P_2_O_13_] units formed along the b- and c-axes are connected to [P_2_O_7_] groups along the a-axis by shared corners or edges. Each [Fe_3_P_2_O_13_] unit comprises three FeO_6_ octahedra and three PO_4_ tetrahedra, together forming three-dimensional channel that facilitates rapid sodium-ion diffusion [22]. Rietveld refinement information reveals that the lattice parameters of NFPP-Ni and NFPP-Mn are slightly larger than pure NFPP, indicating successful introduction of Ni/Mn dopants into the NFPP crystal structure and the resulting lattice expansion. This is due to the larger ionic radii of Ni^2+^ (0.69 Å) and Mn^2+^ (0.67 Å) compared to that of Fe^2+^ (0.61 Å), as well as the influence of Ni^2+^/Mn^2+^ on the Fe−O covalent bonding environment [23, 24].

In addition, the organic chelating agent citric acid and its associated acid radicals underwent pyrolysis during the sintering process, resulting in the generation of an in situ carbon coating on the particle surfaces. The properties and graphitization degree of the surface carbon are further investigated via Raman spectra (Fig. 2d). The three samples correspond to disordered carbon in the D-band and graphitic carbon in the G-band located at 1354 and 1594 cm^−1^. The I_D_/I_G_ values of NFPP, NFPP-Ni, and NFPP-Mn are 0.70, 0.64, and 0.66, respectively. This implies that all three samples possess a well-conductive carbon layer and that Ni/Mn doping catalyzes the graphitization of the surface carbon layer to a certain extent [14]. Furthermore, thermogravimetric (TG) analysis is performed to determine the carbon content in NFPP, NFPP-Ni, and NFPP-Mn, which is found to be 7.62%, 8.82%, and 8.95%, respectively (Fig. S3). The SEM results of all samples show an irregularly stacked bulk morphology, suggesting that Ni/Mn doping does not markedly impact the overall morphology (Fig. S4). The EDS analysis presents uniform distributions of Ni/Mn dopants in the samples. In high-resolution transmission electron microscopy (HRTEM) images (Fig. 2e), the lattice fringe width of NFPP-Ni is 0.37 nm, corresponding to the (410) crystal plane. In contrast, the HRTEM analysis of pure NFPP and NFPP-Mn is illustrated in Fig. S5 [25].

To assess the chemical bonding states and electronic interactions, the three as-prepared samples are further characterized by XPS. The Fe 2*p* XPS spectrum of all samples (Fig. 2f) can be fitted to Fe 2*p*_1/2_ and Fe 2*p*_3/2_ peaks. For NFPP-Ni, the Ni 2*p* signal is distinctly detected (Fig. 2g), further confirming the successful doping of Ni^2+^. Interestingly, the characteristic Fe 2*p*_3/2_ peak associated with Fe^3+^ in NFPP-Ni shifts toward lower binding energies compared to NFPP and NFPP-Mn, implying increased electron density at the Fe sites. This observation suggests that electronic coupling between Fe^3+^ and Ni^2+^ ions effectively modulates the electronic configuration of the Fe in NFPP cathode. In addition, the Ni 2*p* XPS spectrum for NFPP-Ni under different charge/discharge states is presented in Fig. 2g. It is evident that Ni^2+^ ions in the initial state are oxidized to Ni^3+^ during charging and subsequently return to the initial state after complete discharge, demonstrating the ideal electrochemical reversibility of Ni [18]. The local coordination environment of Fe in the materials, along with the occupancy site of the Ni dopant in the NFPP lattice, is investigated using Fourier transformation-EXAFS (FT-EXAFS) and corresponding fitting outcomes. As depicted in Fig. 2h, two peaks appear at 1.5 and 2.5 Å in Fe K-edge k^3^-weighted XANES spectrum, corresponding to the first shell Fe−O path and the second shell Fe−O−Fe path, respectively. Compared with the pure NFPP, both the asymmetry of Fe−O peak and the intensity of Fe−O−Fe peak in NFPP-Ni and NFPP-Mn are decreased, suggesting that Ni and Mn dopants can increase the degree of local distortion in the FeO_6_ octahedron. Notably, the negative shift in the Fe−O−Fe peak position in NFPP-Ni, as highlighted in the inset of Fig. 2h, indicates the presence of electronic interactions between Fe and Ni atoms, distinct from the effects observed for Mn doping. Moreover, the Ni K-edge FT-EXAFS spectrum in R space for NFPP-Ni also exhibits two pronounced coordination peaks at 1.5 and 2.5 Å (Fig. 2i). These peaks are essentially identical to those observed in the Fe K-edge spectrum, suggesting that Ni substitutes for Fe sites within the NFPP host lattice. Figures S7–S9 present the Fe K-edge k^3^χ data of EXAFS oscillations data and fitting curves for the three samples. For NFPP-Ni, the Fe−O bond length is shortened to 2.07 Å, while the Fe−Fe/Ni bond length increases to 3.09 Å. In addition, the fitted coordination numbers (CN) for Fe−O and Fe−O−Fe/Ni in NFPP-Ni are calculated to be 6.0 and 5.0, respectively, both higher than NFPP (Table S6). This structural feature is favorable for accelerating electron transfer during charge–discharge processes, thus improving reaction kinetics. The density functional theory (DFT) calculation reveals that the average Fe−O bond length in NFPP-Ni is reduced by 0.02 Å (Figs. 2j and S6), suggesting that Ni doping induces an appropriate crystal structure distortion in NFPP [26].

### Electron-Coupled Interactions

**Fig. 3:**
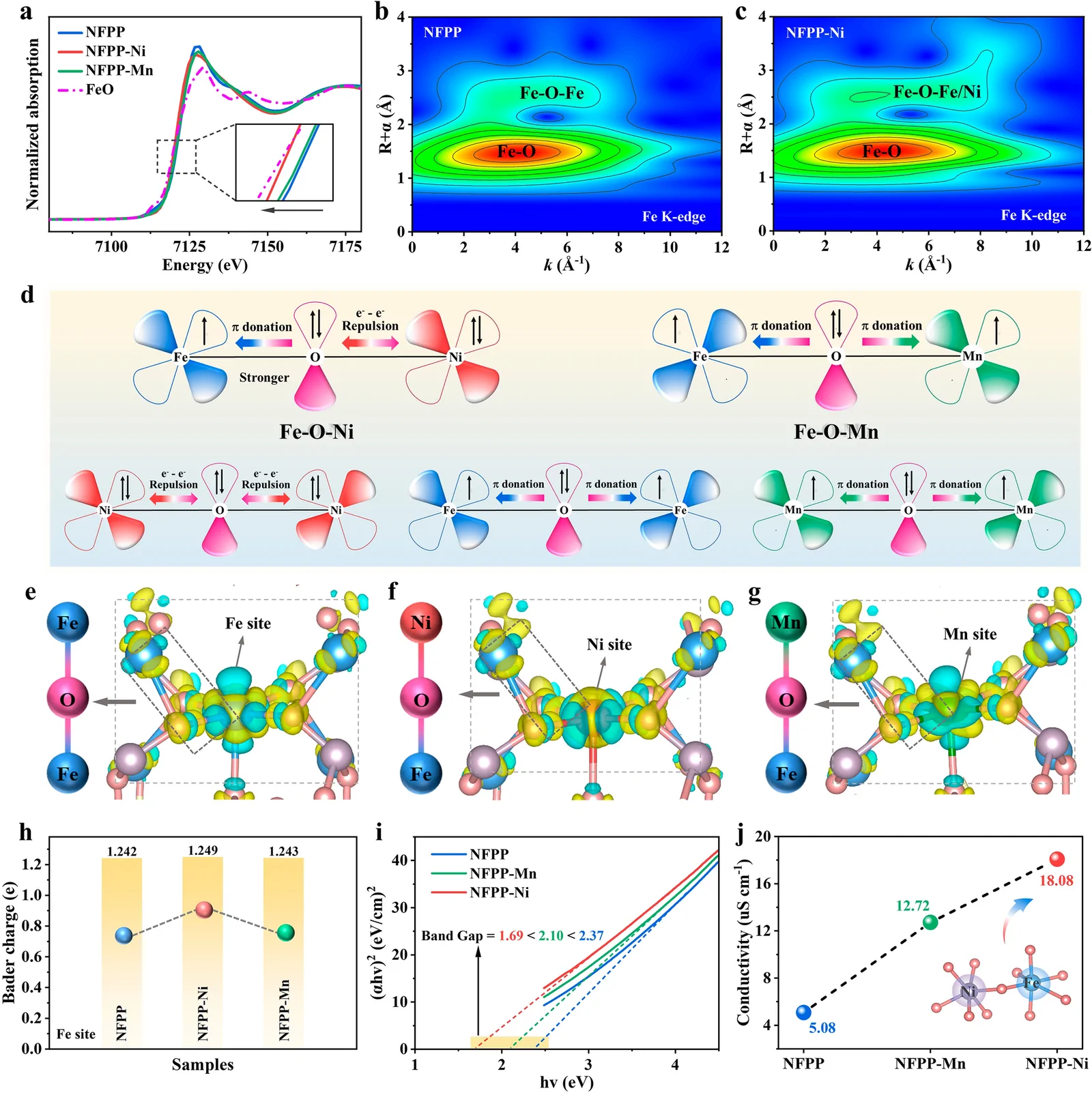
**a** XANES spectra of NFFP, NFFP-Ni, and NFFP-Mn at Fe K-edge. The Fe K-edge wavelet transform (WT)-EXAFS nephograms of **b** NFPP and **c** NFPP-Ni. **d** Illustration of electronic coupling between Fe, Ni, and Mn. Difference charge densities of **e** NFPP, **f** NFPP-Ni, and **g** NFPP-Mn. **h** Bader charge transfers, **i** Tauc plots derived from ultraviolet–visible (UV–Vis) diffuse reflectance spectra, and **j** four-point probe conductivity measurements of NFFP, NFFP-Ni, and NFFP-Mn

The electronic state of Fe was evaluated via XANES (Fig. 3a). The Fe K-edge XANES spectrum of NFPP-Ni exhibits a shift toward lower energy compared to those of pure NFPP and NFPP-Mn, indicating a reduced valence state of Fe and elevated electron density. In contrast, the Ni K-edge XANES spectras largely overlap in NFPP-Ni and NiO, suggesting partial electron transfer between Fe and Ni in NFPP-Ni (Fig. S10) [27, 28]. Wavelet transform (WT)-EXAFS is performed to distinguish the Fe/Ni−O and Fe/Ni−O−Fe/Ni paths (Figs. 3b, c, S11, and S12). The differences observed between the Fe−O and Fe−O−Fe bonds in NFPP and NFPP-Ni indicate that Ni doping alters the Fe−O/Fe−O−Fe coordination environment within the samples. The close similarity of the TM−O−TM (TM = Fe/Ni) paths in the Fe K-edge and Ni K-edge spectra of NFPP-Ni verifies that Ni atoms substitute for Fe sites in the NFPP lattice. Moreover, Fe and Ni in the NFPP-Ni sample prefer to bridge through O to form localized Fe−O−Ni linkages rather than direct Fe−Ni bonds, thereby regulating the electronic structure between Fe and O in the FeO_6_ octahedron [29, 30].

Based on the above XPS and XANES analysis, it is evident that the introduction of Ni ions induces electronic coupling with Fe ions in the NFPP material via the valence electron configurations of Fe, Ni, and Mn and their interactions with the O ligands. Specifically, both Fe^3+^ and Mn^2+^ possess a 3d^5^ (t_2g_^3^ e_g_^2^) electron configuration (Fig. S14). The unpaired electrons in their π-symmetric d orbitals (t_2g_, d_xy_, d_xz_, d_yz_) lead to weak interactions between Fe^3+^/Mn^2+^ and the bridging O^2−^ through the π-donation. By comparison, Ni^2+^, with a fully occupied π-symmetric d orbital (3d^8^, t_2g_^6^ e_g_^2^), experiences strong electron–electron repulsion with O^2−^ [31, 32]. The electronic interactions involving Fe^3+^ and Ni^2+^/Mn^2+^ in NFPP-Ni and NFPP-Mn are analyzed using Fe−O−Mn and Fe−O−Ni units (Fig. 3d). Upon the coupling of Fe^3+^ and Ni^2+^, the enhanced electron repulsion at the Ni − O bond promotes π-donation between Fe and O, thus promoting charge transfer from Ni^2+^ to Fe^3+^. Additionally, Ni^2+^ ions act as an intermediate electron bridge along the Fe−O−P−O−Fe pathway, accelerating the electron transfer. Conversely, Mn^2+^, which bridges in a manner similar to Fe^3+^, does not produce this effect [27]. Theoretical calculations further support these electron transfer processes. The differential charge density diagrams (Fig. 3e-g) illustrate clear distinctions in charge redistribution among NFPP, NFPP-Ni, and NFPP-Mn, demonstrating that Fe−Ni coupling modulates the local electronic structure and thus optimizes electron transport pathways in the NFPP host [33]. The above analyses indicate that, in Ni-doped NFPP, partial charge transfer occurs from Ni to Fe via oxygen bridge (O^2−^) between the metal ions, which is corroborated via Bader charge analysis for Fe, Ni, and Mn (Figs. 3h and S13). Overall, both experiments and density functional calculations have revealed an increase in the electron density at the Fe position.

To elucidate the influence of ion incorporation on the electrical conductivity, the band gap energy and electronic conductivity of samples are evaluated using UV–Vis spectrum and four-point probe. The band gap energies, determined from the intersection of the tangent line with the photon energy (h*v*) axis, are 2.37 eV for NFPP, 1.69 eV for NFPP-Ni, and 2.10 eV for NFPP-Mn (Figs. 3i and S15). The narrower band gap in the NFPP-Ni sample correlates with its higher electrical conductivity [34, 35]. Four-point probe measurements also show that the electronic conductivity of NFPP-Ni is noticeably superior than NFPP and NFPP-Mn (Figs. 3j, S16, and Table S7). All the results collectively indicate that the incorporation of Ni^2+^ ions more efficiently modulates the electronic structure of the NFPP, thereby enhancing conductivity, which is expected to promote electron transport and Na^+^ diffusion under high-rate conditions.

### Electrochemical Behaviors

**Fig. 4:**
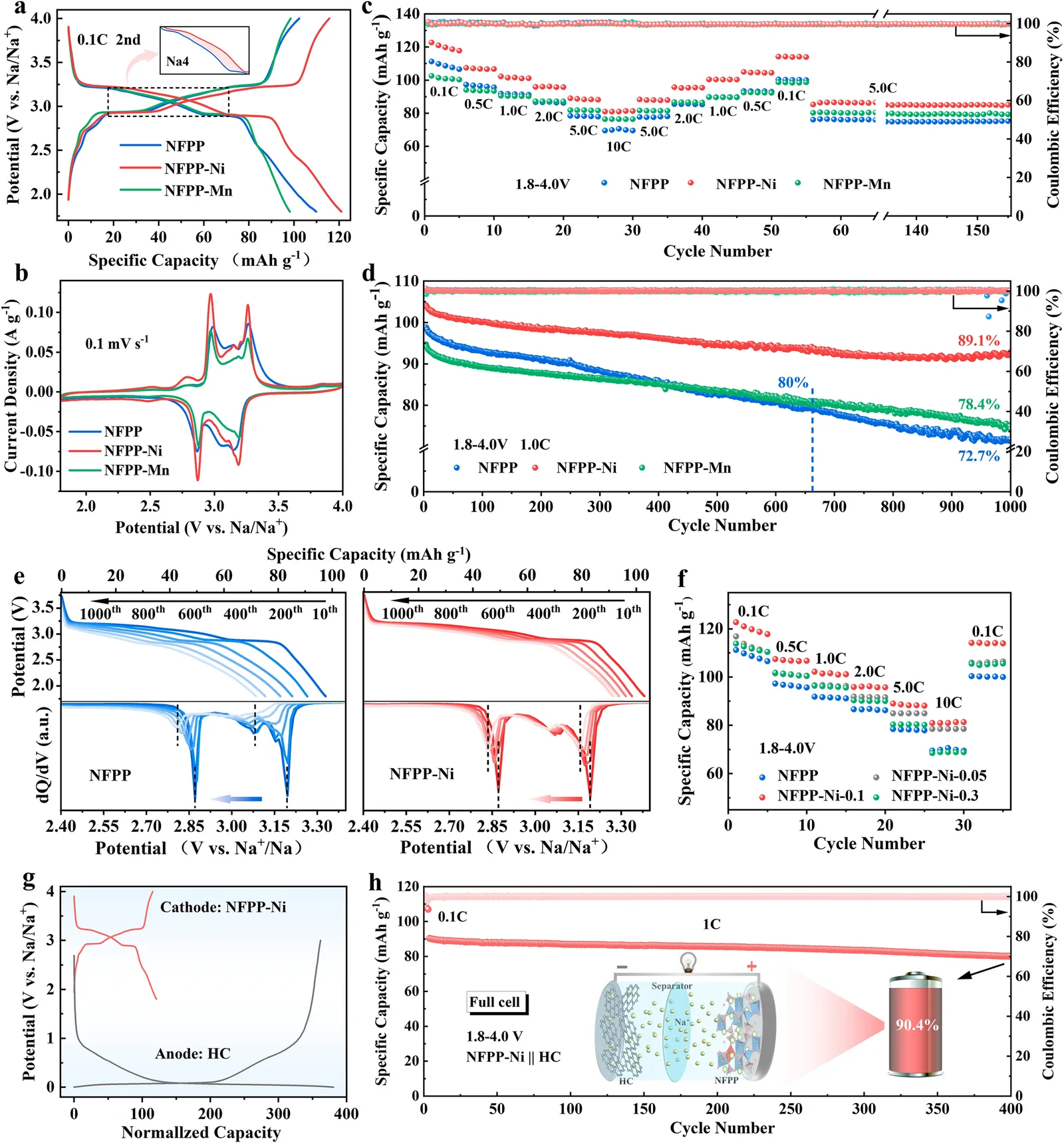
**a** The GCD curves at 0.1C of NFFP, NFFP-Ni and NFFP-Mn. **b** CV profiles of NFFP, NFFP-Ni, and NFFP-Mn. The rate capability from 0.1 to 10C for **c** NFFP, NFFP-Ni, and NFFP-Mn, and **f** NFPP, NFFP-Ni-0.05, NFFP-Ni-0.1, and NFFP-Ni-0.3. **d** Cycling performance over 1000 cycles at 1C for NFFP, NFFP-Ni and NFFP-Mn. **e** Discharge curves and corresponding dQ/dV plots of NFPP and NFPP-Ni at different cycle numbers (from the 10th to the 100th cycle). **g** The GCD profiles of NFPP-Ni and HC electrode in half cell. **h** The cycling performance of NFPP-Ni||HC full cell

Figure 4a-b displays galvanostatic charge–discharge (GCD) curves and cyclic voltammetry (CV) profiles for NFPP, NFPP-Ni, and NFPP-Mn. It is evident that multiple plateaus observed in the GCD curves correspond closely to the redox peaks in the CV profiles, which are associated with Na^+^ insertion/extraction at different sites in the NFPP material, as reported previously [11]. The NFPP-Ni electrode delivers a specific capacity of 121.01 mAh g^−1^, which is higher than NFPP (109.83 mAh g^−1^) and NFPP-Mn (101.43 mAh g^−1^). Notably, this capacity enhancement in NFPP-Ni relative to NFPP and NFPP-Mn is concentrated within the 2.9–4.0 V. This may cause by the electron-coupled interactions between Ni^2+^ and Fe^3+^, which accelerate electron transfer and promote greater Na^+^ extraction from the Na4 sites. In contrast, the introduced Mn^2+^ ions, due to the different electronic structure from Ni^2+^, did not cause an increase in the capacity of NFPP resulting from electronic coupling. Furthermore, the CV profiles reveal that NFPP-Ni exhibits sharper and more symmetrical oxidation/reduction peaks with a larger enclosed area compared to the other two electrodes. This proves that Ni^2+^ doping effectively reduces the intrinsic polarization of the NFPP material and expedites Na^+^ reaction kinetics.

The rate capability at varying current densities is shown in Fig. 4c. The NFPP-Ni cathode delivers specific discharge capacities of 121.0, 107.4, 102.2, 95.8, 89.0, and 80.9 mAh g^−1^ at 0.1, 0.5, 1, 2, 5, and 10C, respectively, which are substantially higher than NFPP and NFPP-Mn. These results demonstrate that Ni^2+^ doping markedly improves the rate capability. Besides, as can be seen in Fig. S17, the electrochemical property of NFPP-Ni surpasses that of many previously reported polyanionic NFPP cathode for SIBs. The cycling performance of NFPP, NFPP-Ni, and NFPP-Mn cathodes is shown in Fig. 4d. After 1000 cycles at 1C, NFPP-Ni exhibits a specific discharge capacity of 92.8 mAh g^−1^, corresponding to 89.1% capacity retention. For comparison, NFPP and NFPP-Mn exhibit similar capacity retention after only 305 and 450 cycles, respectively. The resulting dQ/dV curves under different cycle numbers further reveal that the reduction peak displacement of NFPP-Ni is the smallest (35.9 mV) from the 10th to the 1000th cycle, while those of NFPP and NFPP-Mn are 61.7 and 96.4 mV, respectively (Figs. 4e and S18) [36]. These findings together validate the enhanced structural stability and redox reversibility of the NFPP-Ni electrode. On this basis, the impact of Ni doping amount on the property of NFPP is explored. As displayed in Figs. 4f and S19, the sample with a doping level of 0.1 (NFPP-Ni-0.1) possesses the best rate capability and cycling stability. Evidently, introducing an appropriate amount of Ni^2+^ at the Fe^2+^ sites promotes electron repulsion within the Fe−O−Ni units, thereby accelerating electron transport and facilitating rapid redox reactions. Meanwhile, the strengthened Fe−O covalent bonding helps stabilize the NFPP crystal structure, leading to excellent electrochemical properties (as detailed in Fig. 6a).

Next, the practical application of NFPP-Ni is evaluated by assembling a full cell (denoted as NFPP-Ni||HC). The GCD curves of the NFPP-Ni and hard carbon (HC) in their respective half cells are shown in Fig. 4g. The cycling performance of the HC is presented in Fig. S20. The GCD curves and rate performance of NFPP-Ni||HC are displayed in Fig. S21a-b. The specific capacities of full cell are 109.90, 105.49, 100.07, 96.44, 91.73, and 83.73 mAh g^−1^ at 0.1, 0.2, 0.5, 1.0 2.0, and 5.0C, respectively. What is more, it exhibits impressive long-term Na^+^ storage capability, with a capacity retention of 90.4% after 400 cycles at 1C (Fig. 4h). The above results underscore the considerable promise of NFPP-Ni for practical SIB applications.

### Electrochemical Kinetics

**Fig. 5:**
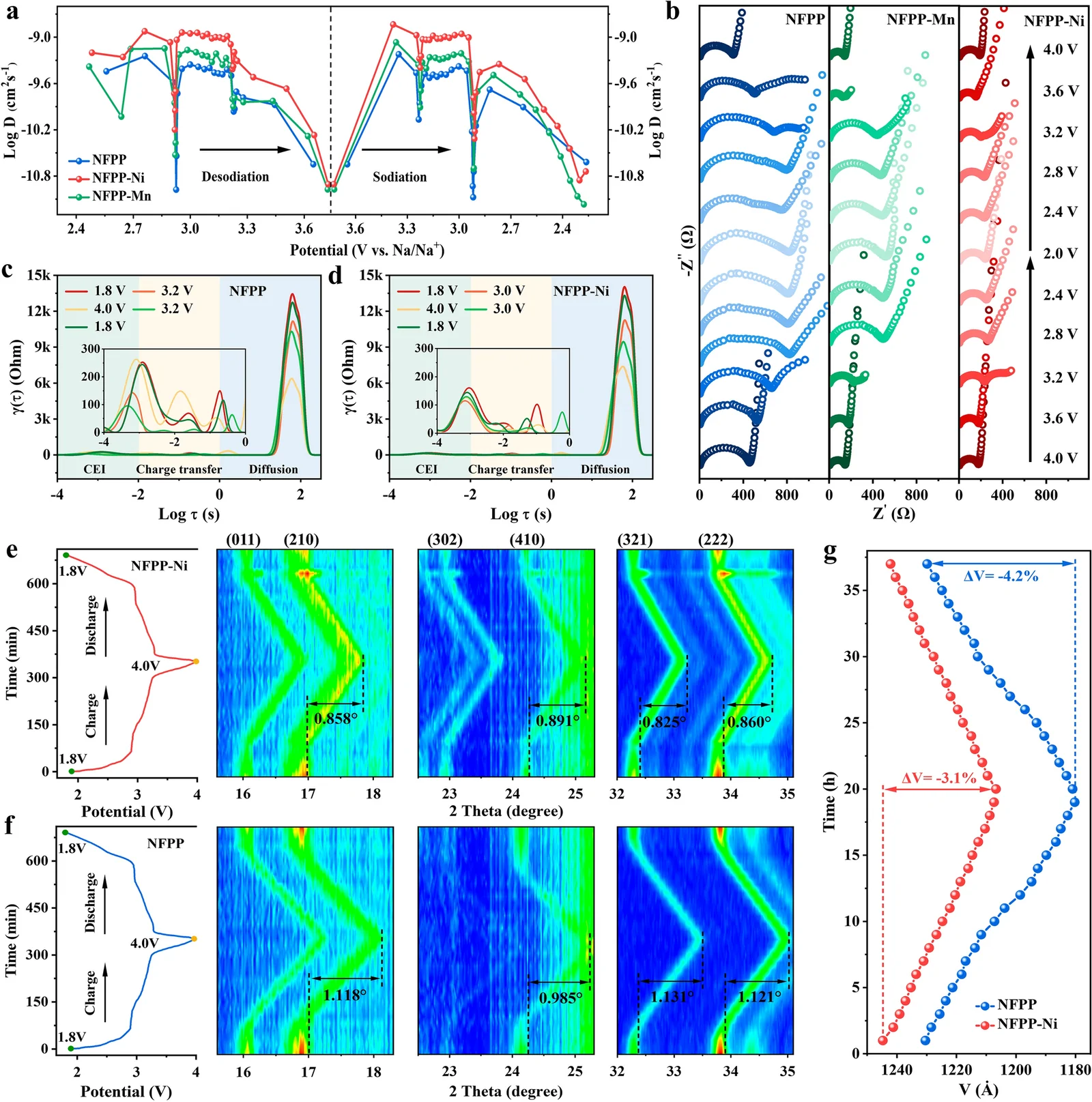
**a** Corresponding log D plots during desodiation and sodiation for NFPP, NFPP-Ni, and NFPP-Mn. **b** In situ EIS spectra. The DRT analysis of **c** NFPP and **d** NFPP-Ni. In situ XRD of **e** NFPP-Ni and **f** NFPP. **g** Lattice volume change upon Na^+^ insertion/extraction

To further examine the Na^+^ ion diffusion behavior of NFPP, NFPP-Ni, and NFPP-Mn, a series of kinetic measurements are taken. The CV tests at various scan rates were performed on all samples to qualitatively compare the sodium diffusion capacity across the four samples and to evaluate the impact of Ni doping on diffusion rates (Fig. S22). Figure S23 presents the CV curves and the linear relationships between peak current (*i*_*p*_) and the square root of the scan rate (*v*^1/2^). As the scan rate rises, the currents of oxidation (O1/O2) and reduction (R1/R2) peaks increase significantly. Among the three samples, the NFPP-Ni electrode shows the smallest increase in the potential difference between the O1/O2 and R1/R2 peaks, as well as a steeper slope in the *i*_*p*_-*v*^1/2^ plot, indicating that a small degree of polarization is a fast reaction rate. Based on the slopes (b-values) derived from the log (*i*_*p*_) versus log (*v*) plots (Fig. S24), the b-values for NFPP-Ni are 0.61, 0.75, 0.68, and 0.76, suggesting that the charge storage mechanism is dominated via both Faradaic and non-Faradaic processes. Furthermore, Fig. S25 shows that when the scan rate increases from 0.1 to 1.0 mV s^−1^, the capacitive contribution of NFPP-Ni increases from 82 to 94%, which is higher than that of NFPP (from 76% to 91%) and NFPP-Mn (from 79% to 92%) samples. This is responsible for the fast kinetics achieved via Ni doping [11]. In the continuous galvanostatic intermittent titration technique (GITT) profiles illustrated in Fig. S26, the NFPP-Ni sample presents a lower overpotential, indicating a smaller degree of polarization. The log D values (where D is the Na^+^ diffusion coefficient) for all samples vary between 10^−9^ and 10^−11^ cm^2^ s^−1^, with all three displaying similar trends. Particularly, the log D values for NFPP-Ni are generally higher than NFPP and NFPP-Mn (Fig. 5a). The average Na^+^ diffusion coefficients of NFPP, NFPP-Ni, and NFPP-Mn are 7.43E − 10, 1.96E − 9, and 1.02E − 9 cm^2^ s^−1^, respectively, implying that the incorporation of Ni significantly enhances Na^+^ diffusion in the NFPP material (see calculation details in Fig. S26).

The electrochemical impedance spectroscopy (EIS) spectra and corresponding fitted resistance values (Fig. S27 and Table S8) indicate that the NFPP-Ni sample exhibits the smallest semicircle in the high-frequency region and the lowest linear fitting coefficient (σ) in the low-frequency region, reflecting the most rapid charge transfer among the samples. The equivalent circuit used for fitting (inset, Fig. S27) is identical for all samples. In situ EIS analysis of NFPP, NFPP-Ni, and NFPP-Mn electrodes is demonstrated in Fig. 5b. All three samples display similar trends during the charge–discharge process. Interestingly, NFPP-Ni shows a smaller charge transfer resistance (R_ct_), which remains largely stable throughout cycling (Fig. S29). This indicates that NFPP-Ni possesses excellent reversibility while maintaining minimal Na^+^ diffusion resistance [37]. Further insight is provided by distribution of relaxation times (DRT) analysis of in situ EIS spectrum (Figs. 5c-d and S28). In general, the intervals of Log τ < -2 s, -2 s < Log τ < 0 s, and Log τ > 0 s correspond to the cathode–electrolyte interface (CEI), R_ct_, and ion diffusion processes, respectively. The CEI and R_ct_ features for NFPP-Ni are associated with smaller τ values and stronger Na^+^ ion diffusion, revealing the superior rate capability achieved through Ni^2+^ doping [38, 39].

In situ XRD profiles of NFPP and NFPP-Ni electrodes are collected during electrochemical reaction process (Fig. S30), revealing the structural evolution of the cathodes during desodiation/sodiation. The diffraction peaks shift regularly, with no new peaks appearing or original peaks disappear throughout the electrochemical process, suggesting a highly reversible and stable solid-solution reaction mechanism [40]. Figure 5e-f provides enlarged views of selected indexed peaks with noticeable variation in NFPP-Ni and NFPP. Upon charging from 1.8 to 4.0 V, the (011), (210), (302), (410), (321), and (222) planes of both samples move to higher 2θ values, signifying lattice contraction, and then return reversibly to original positions during Na^+^ insertion. Notably, the indexed peaks of the NFPP-Ni electrode display more moderate intensity changes and smaller shifts in peak position during Na^+^ insertion/extraction compared to NFPP. This suggests that NFPP-Ni facilitates a faster Na^+^ migration rate with less lattice strain, allowing for greater Na^+^ insertion/extraction with smaller volume changes [41]. As shown in Fig. S30, the lattice parameters (a, b, and c) all decreased during the Na^+^ extraction and then increase during the Na^+^ insertion. It can be observed that the variation range of NFPP-Ni is smaller than that of NFPP. The resulting lattice volume changes are 4.2% for NFPP and 3.1% for NFPP-Ni (Fig. 5g), confirming that the NFPP-Ni electrode maintains a more robust crystal framework under long-term cycling and high-rate conditions. This mainly stems from two synergistic mechanisms: (1) The electron coupling between Ni^2+^ and Fe^3+^ can be utilized to mitigate the dynamic barriers to Na^+^ diffusion; (2) The appropriate Fe−O covalent degree induced by Ni^2+^ alleviates the crystal stress during the Na^+^ migration process. Overall, adjusting the electronic structure and crystal structure can achieve remarkable rate capability and cycling stability.

### Modulation Mechanism of Fe−O Covalency by e_g_ Orbital Occupancy

**Fig. 6:**
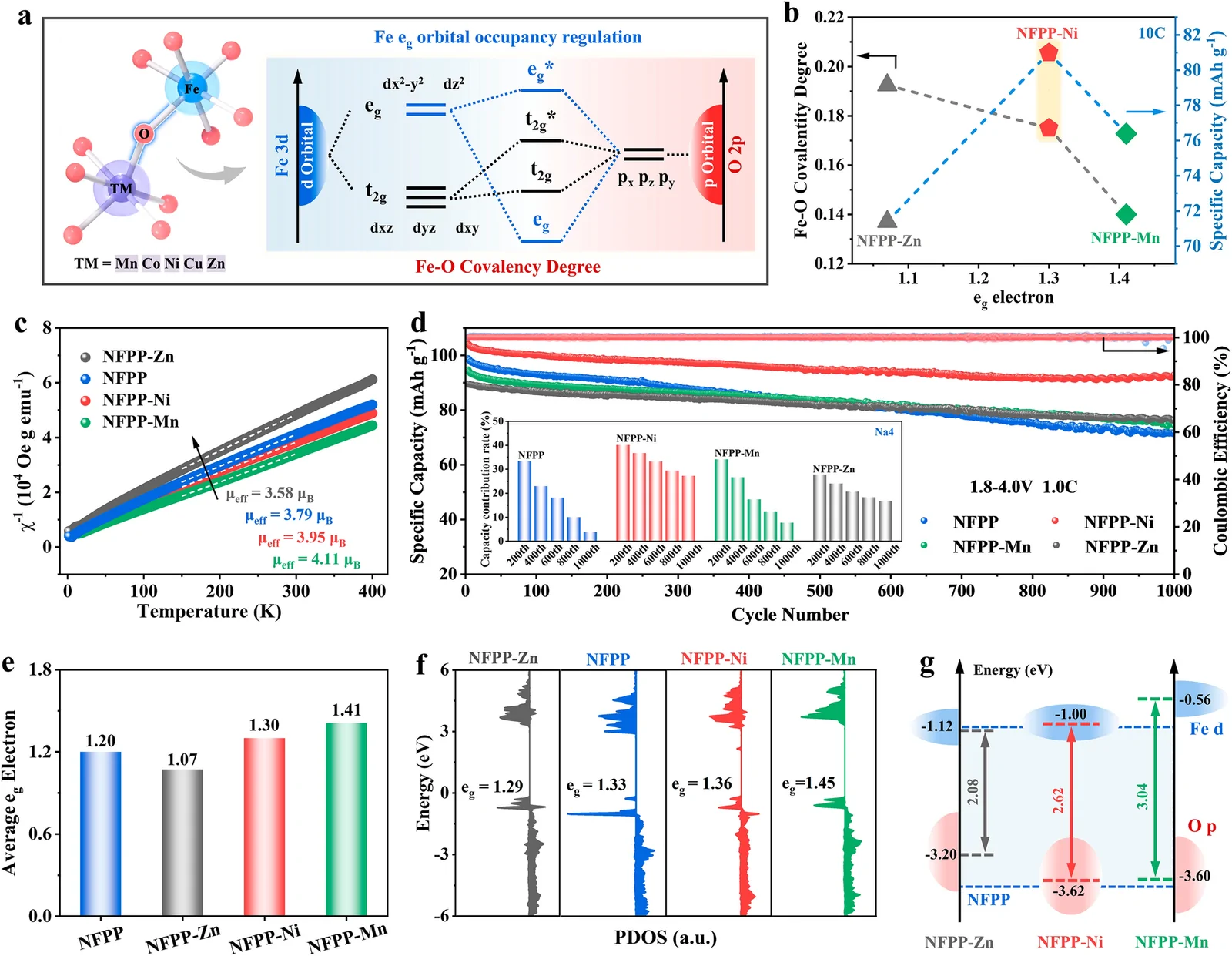
**a** Schematic illustration of Fe−O covalency modulation by e_g_ orbital occupancy, affecting d−p orbital hybridization. **b** Relationship among Fe−O covalency, specific capacity at 10C, and e_g_ electron count. **c** Temperature-dependent inverse magnetic susceptibility (1/χ) fitted using the Curie–Weiss law. **d** Cycling performance and Na4-site contribution (inset c) for NFPP, NFPP-Zn, NFPP-Ni, and NFPP-Mn. Values of e_g_ orbital occupancy calculated by **e** M-T measurements and **f** PDOS analysis. **g** Energy gap between the Fe d and O p band centers

Based on the above analyses, it is evident that complete or partial occupation of the t_2g_ orbitals of M^n+^ ions leads to different bridging modes with O^2−^, thereby affecting the electron transfer behavior at the redox center. Given that Cu and Zn share the same t_2g_ orbital occupancy as Ni, there remains some debate regarding the underlying mechanism of electrochemical activity enhancement in NFPP cathodes [42, 43]. To address this, a series of TM elements (Mn/Co/Ni/Cu/Zn) is introduced into the NFPP material (Fig. 6a). It is found that these TM elements can optimize the interaction between the Fe d band and O p band (i.e., the Fe−O covalency degree) by modulating the e_g_ orbital occupancy of Fe^2+^. This, in turn, enhances electron transfer at high rates, improves ion transport, and prolongs the cycling lifetime of NFPP-based materials. As indicated in Fig. 6c, the χ^−1^-T curves obtained from temperature-dependent magnetic susceptibility (M-T) measurements (Fig. S34) are fitted over the range of 150–300 K to determine the effective magnetic moments (μ_eff_) for NFPP-Zn, NFPP, NFPP-Ni, and NFPP-Mn [44, 45]. Using the equations provided in Fig. S33, the e_g_ occupancy values for NFPP-Zn, NFPP-Ni, and NFPP-Mn are calculated as 1.07, 1.30, and 1.41, respectively (Fig. 6e). Interestingly, DFT calculations analyzing partial density of states (PDOS) for the e_g_ electrons of Fe in these samples (Fig. 6f) uncover the same trend. That is, the filling of e_g_ electrons on Fe d orbital induced by the fully occupied t_2g_ orbitals of Ni^2+^ (1.36) is intermediate between those induced by Zn^2+^ (1.29) and by Mn^2+^ (1.45).

As illustrated in Fig. 6g and Table S9, the Fe d and O p band centers are obtained by integrating the PDOS of Fe and O in Fig. S32 and S33. It is well established that the energy difference between TM d band center and O p band center reflects the TM−O covalency degree, with a smaller energy difference demonstrating the stronger TM−O covalent interaction [46]. The results show that the energy differences between Fe d and O p band centers in NFPP-Zn, NFPP-Ni, and NFPP-Mn are 2.08, 2.62, and 3.04, respectively. As illustrated in Fig. 6b, a volcano-type relationship is observed between the e_g_ occupancy and Fe−O covalency (Table S10) in the NFPP samples with different TM^n+^ dopants. Obviously, the e_g_ orbital occupancy at the Fe sites regulates the interaction between the Fe d and O p bands. This can be attributed to the crystal field splitting in the FeO_6_ octahedral environment (Fig. 6a), where the Fe d orbitals are divided into e_g_ (d_z_^2^, d_x_^2^_-y_^2^) and t_2g_ (d_xy_, d_xz_, d_yz_) subsets. Unlike the t_2g_ orbitals, the e_g_ orbitals point directly toward the ligand O, resulting in greater spatial overlap with the O p orbitals (Fig. S32). In the case of NFPP-Mn, the higher e_g_ electron count increases the Coulomb repulsion between Fe d and O p electrons, thereby weakening the electron sharing between Fe and O and reducing the covalency of Fe−O bond [47].

The degree of TM−O covalency may also be a critical factor in governing the electrochemical kinetics of cathode [48, 49]. The rate and cycling performances of NFPP, NFPP-Mn, NFPP-Co, NFPP-Ni, NFPP-Cu, and NFPP-Zn cathodes are indicated in Figs. 6d, S35 and S36. Notably, NFPP-Ni stands out with the highest specific discharge capacity at a high rate of 10C, as well as excellent cycling stability (Fig. S35). Importantly, the relationship between the e_g_ occupancy of Fe and the high-rate electrochemical activity of the TM^n+^-doped NFPP samples also shows a volcano-type trend (Fig. 6b), paralleling the Fe−O covalency relationship. In specific, NFPP-Ni, with an e_g_ orbital occupancy of 1.30 for Fe, achieves moderate Fe−O covalency and delivers the highest specific discharge capacity among the series. On the basis of the classical “Sabatier principle” in the field of electrocatalysis, an ideal catalyst should exhibit neither too strong nor too weak binding to reaction intermediates [43]. Analogously, for electrode materials, the covalency between the redox center TM and O should also be moderate. In the NFPP system, the dopant TM element modulates the overlap between Fe d and O p orbitals by altering the e_g_ orbital occupancy at the active Fe site. The resulting Fe−O bond strength directly impacts the reversibility of Na^+^ migration and the rate of electron transfer at high current densities. In general, a lower M−O covalency degree implies that the electron cloud of M is less tightly bound to O, making M^n+^ ions more prone to losing or gaining electrons during redox reactions and thereby accelerating electron transfer. However, this also weakens the covalent bond between M and O, which may result in progressive distortion and structural degradation of crystal lattice during repeated ion intercalation/deintercalation. Compared with NFPP-Zn and NFPP-Mn, the NFPP-Ni sample exhibits the optimal e_g_ orbital occupancy at the Fe sites and the most moderate degree of Fe−O covalency, as tuned by Ni^2+^ doping. This configuration provides stable migration channels and maximizes Na4-site utilization for reversible ion transport under long-term cycling. Simultaneously, due to the lower conduction band position, the band gap of NFPP-Ni is reduced and its intrinsic electronic conductivity is enhanced, which can facilitate efficient charge transfer at the Fe sites during high-rate “sodiation-desodiation” processes.

## Conclusion

In summary, the electronic structure of Fe^3+^ is ingeniously reconstructed via introducing Ni^2+^, forming an “electron transfer expressway” in which Ni^2+^ acts as an intermediate electron bridge that effectively alleviates the sluggish electron transport in NFPP cathodes. Both XANES and DFT results indicate that the electronic coupling effect between Ni^2+^ and Fe^3+^ accelerates electron transfer, while the shortening of Fe−O bonds enhances crystal structure stability. Furthermore, Ni^2+^ doping modulates the electron configuration (e_g_ orbital occupancy) of Fe^3+^, enabling precise regulation of Fe−O covalency to achieve a balance between rate capability and cycling stability. Compared to NFPP, FPP-Mn, NFPP-Co, NFPP-Cu, and NFPP-Zn, the NFPP-Ni cathode exhibits an intermediate e_g_ occupancy for the Fe d orbitals induced by Ni^2+^, which corresponds to an optimal covalency with the O p orbitals. Besides, in situ XRD results demonstrate that NFPP-Ni substantially mitigates lattice strain during charge–discharge cycling while augmenting Na^+^ diffusion kinetics. Consequently, the NFPP-Ni electrode realizes an outstanding specific capacity (121.0 mAh g^−1^ at 0.1C) and stable cyclability (89.1% capacity retention after 1000 cycles). This work provides new insights for the design of advanced iron-based phosphate cathode materials in SIBs, which may also offer reference for the development of other electrodes.

## Supplementary Information

Below is the link to the electronic supplementary material.

Supplementary file1 (DOCX 34404 KB)
