# Supplementary material for: Tailoring eg Orbital Occupancy of Fe in Ni-Doped Na4.3Fe3(PO4)2P2O7 Cathode for High-Performance Sodium-Ion Batteries
Source: Nanomicro Lett. 2026 Feb 5;18:237. doi: 10.1007/s40820-026-02073-3 (PMC12876527; doi:10.1007/s40820-026-02073-3)
Supplement: Supplementary file 1 — Supplementary file1 (DOCX 34404 KB) [file 40820_2026_2073_MOESM1_ESM.docx]

**Tailoring e_g_ Orbital Occupancy of Fe in Ni-Doped Na_4.3_Fe_3_(PO_4_)_2_P_2_O_7_ Cathode for High-Performance Sodium-Ion Batteries**

Xiaoxue Wang^1, 2^, Yuhui Xu^1, 2^, Jianhua Zhang^1, 2^, Yukun Xi^1, 2^, Ningjing Hou^1, 2^, Yixuan Chen^1, 2^, Dongzhu Liu^1, 2^, Zihao Yang^1, 2^, Haocheng Wen^1, 2^, Jia Kang^1, 2^, Xiaoli Yang^1, 2^, Xuexia Song^1, 2^, Jingjing Wang^1, 2^, Wenbin Li^1, 2^, Jiujun Zhang^1, 2, 3,^ *, Kun Zhang^4, 5,^ *, Xifei Li^1, 2,^ *

^1^ Institute of Advanced Electrochemical Energy & School of Materials Science and Engineering, Xi'an University of Technology, Xi'an, Shaanxi 710048, China.

^2^ Shaanxi Engineering Research Center of Key Materials for Lithium/Sodium-ion Batteries, Xi’an University of Technology, Xi'an, Shaanxi 710048, China.

^3^ Institute of New Energy Materials and Engineering, College of Materials Science and Engineering, Fujian Engineering Research Center of High Energy Batteries and New Energy Equipment & Systems, Fuzhou University, Fuzhou, Fujian 350108 China.

^4^ GEM Co., Ltd., Shenzhen, Guangdong 518101, China.

^5^ Hubei Provincial Key Laboratory of High-Value Utilization of Retired Power Batteries, Jingmen, Hubei 448000, China.

*Corresponding authors: jiujun.zhang@fzu.edu.cn (J. Zhang); zhangkun@gem.com.cn (K. Zhang); xﬂi@xaut.edu.cn (X. Li)

*Experimental Section*

**Electrochemical measurements**

Samples of the electrochemical active materials were mixed with Kochen black and polyvinylidene fluoride (PVDF) binder with a weight ratio of 8:1:1 in N-methyl-2-pyrrolidone (NMP) solvent. Then, the electrode slurry was uniformly coated on an Al foil, followed by a drying process in a vacuum oven at 90 °C overnight. Circular working electrodes with a diameter of 12 mm were punched from the Al foil. The electrode was assembled into coin cell (CR2032) using sodium metal as a counter electrode in a glovebox filled with Argon gas. 1 M NaClO_4_ in ethylene carbonate (EC)-propylene carbonate (PC) solution (1:1 by vol.) with 5 vol.% addition of fluoroethylene carbonate (FEC) as electrolyte. The glass fiber paper was used as separator for the Na-ion half-cell. The commercial hard carbon (HC) utilized in this work was purchased from Canrd. Before assembling of Na-ion full cells, the HC anode was pre-sodiumed in half-cell with sodium as counter electrode, and run at 0.2 A g^–1^ within 0.01-3.0 V. The full cells were measured within the electrolyte of 1 M NaClO_4_ in ethylene carbonate (EC)-propylene carbonate (PC) solution (1:1 by vol.) with 5 vol.% addition of fluoroethylene carbonate (FEC) was adopted. The loading of NFPP-Ni active material is 0.72-0.76 mg/cm^2^ (based on the mass of active material per single side). The loading of hard carbon active material is 0.44-0.49 mg/cm^2^ (based on the mass of active material per single side). The cathode current collector is aluminum foil with a thickness of approximately 15 μm; the anode current collector is copper foil with a thickness of approximately 10 μm. The thickness of the cathode active layer is approximately 100 μm (including binder and conductive additive); the thickness of the anode active layer is approximately 100 μm (including binder and conductive additive). A glass fiber separator (Whatman GF/D) with a thickness of approximately 80 μm was used. The pre-sodiated anode was subsequently paired with the NFPP-Ni cathode in a full-cell configuration, cycled between 1.8-4.0 V with an anode-to- cathode capacity (N/P) ratio of 1.2–1.4. Cyclic voltammetry (CV) electrochemical impedance spectroscopy (EIS), and in situ EIS were conducted using a Princeton ParSTAT MC 2000A electrochemical station. The potential range of CV and in situ EIS were 1.8-4.0 V. The amplitude of the signal was set to 5.0 mV with a frequency range of 100 kHz-0.01 Hz. Galvanostatic charge‒discharge (GCD) measurements and the galvanostatic intermittent titration technique (GITT) were performed on a multichannel battery testing system (Neware). All tests were performed at room temperature.

**Theoretical calculation**

All the calculations are performed in the framework of the density functional theory with the projector augmented plane-wave method, as implemented in the Vienna ab initio simulation package.^[1-2]^ The generalized gradient approximation proposed by Perdew-Burke-Ernzerhof (PBE) is selected for the exchange-correlation potential.^[3-4]^ The cut-off energy for plane wave is set to 480 eV. The energy criterion is set to 10^−4^ eV in the iterative solution of the Kohn-Sham equation. The Brillouin zone integration is performed using a 1×4×2 k-mesh. All the structures are relaxed until the residual forces on the atoms have declined to less than 0.05 eV/Å. Calculations were performed using the spin-dependent GGA plus Hubbard U correction. The U values applied were 4.3 eV for Fe, 4.5 eV for Mn, and 6.0 eV for Ni. Data analysis and visualization are carried out with the help of VASPKIT code and VESTA.^[5-6]^


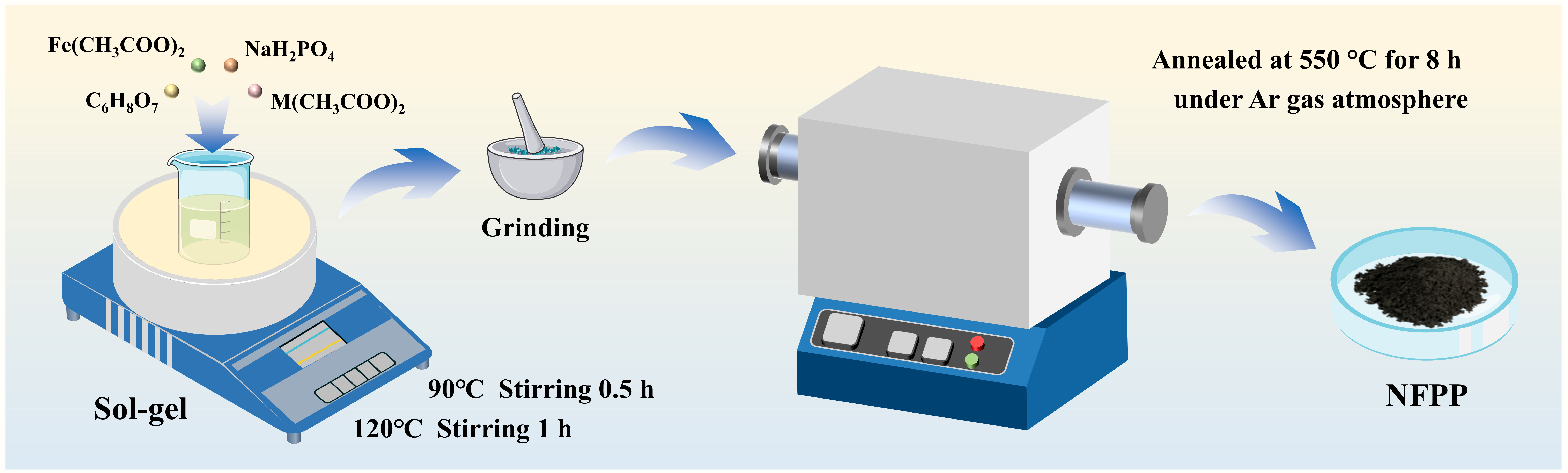


**Fig. S1** The schematic illustration of the synthesis of NFPP, NFPP-Ni, NFPP-Mn, and NFPP-Zn samples.


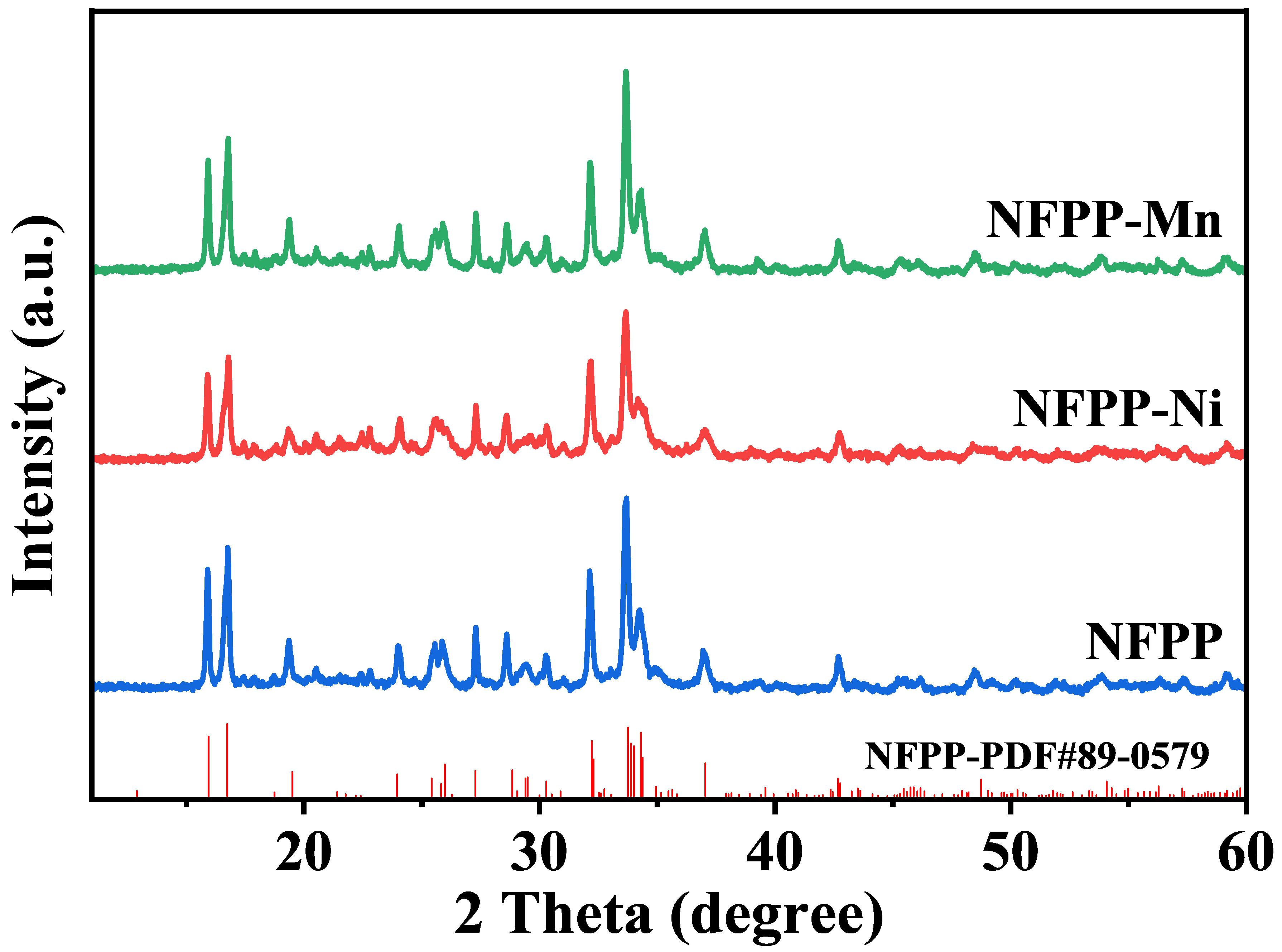


**Fig. S2** XRD patterns of NFPP, NFPP-Ni and NFPP-Mn samples.


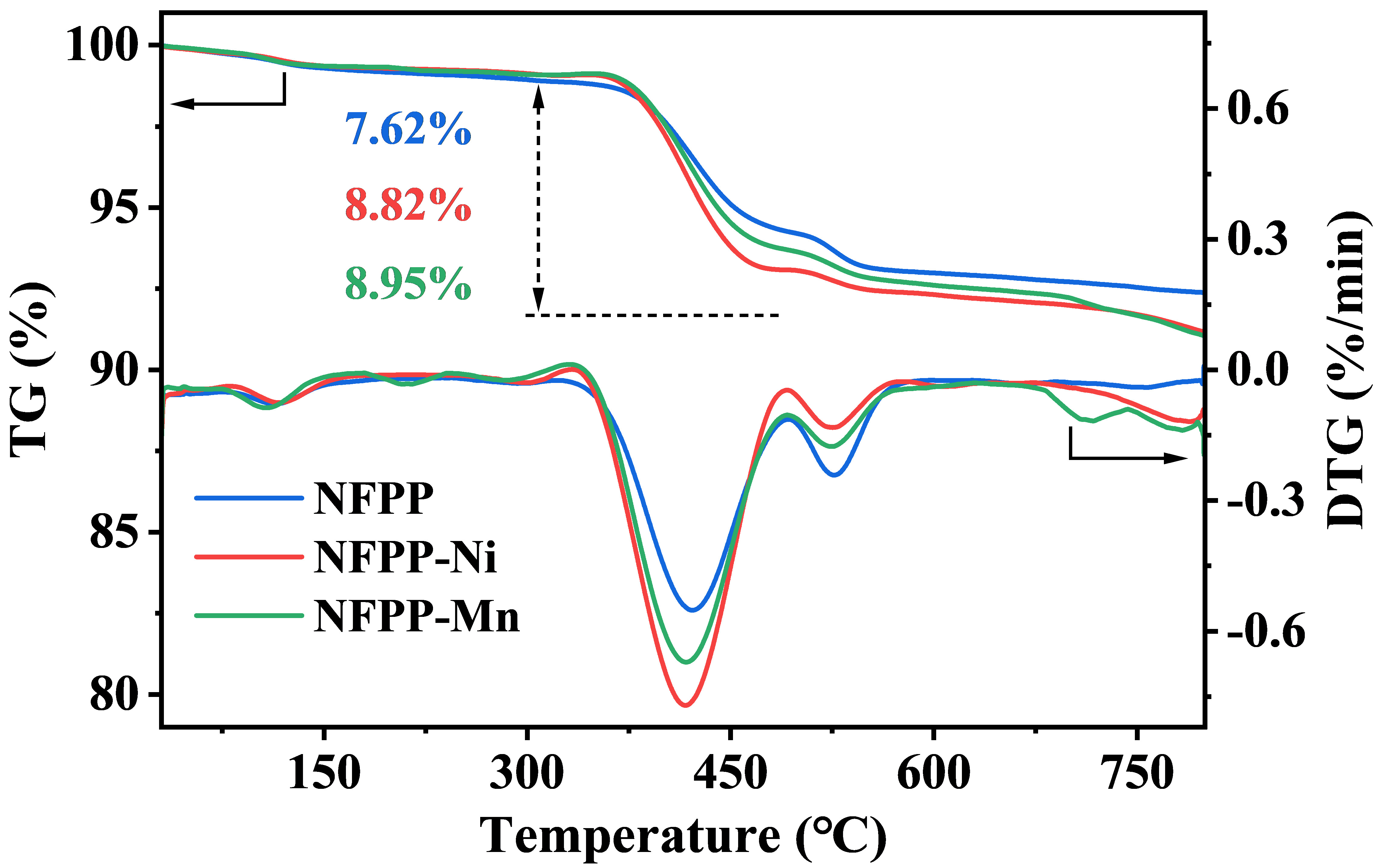


**Fig. S3** TG and DSC curves of NFPP, NFPP-Ni and NFPP-Mn samples.


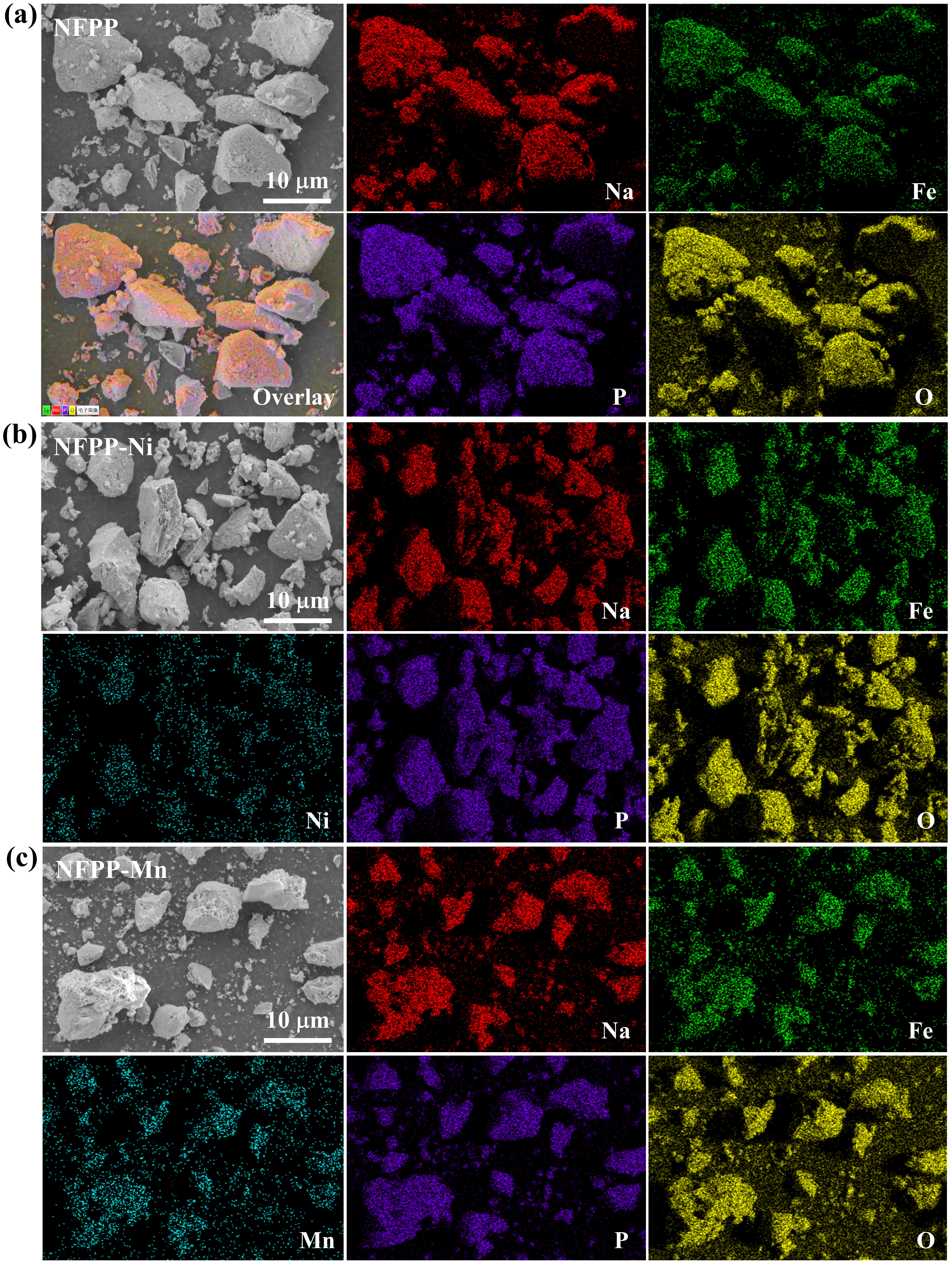


**Fig. S4** SEM image and corresponding EDS mapping images of **a** NFPP, **b** NFPP-Ni and **c** NFPP-Mn.


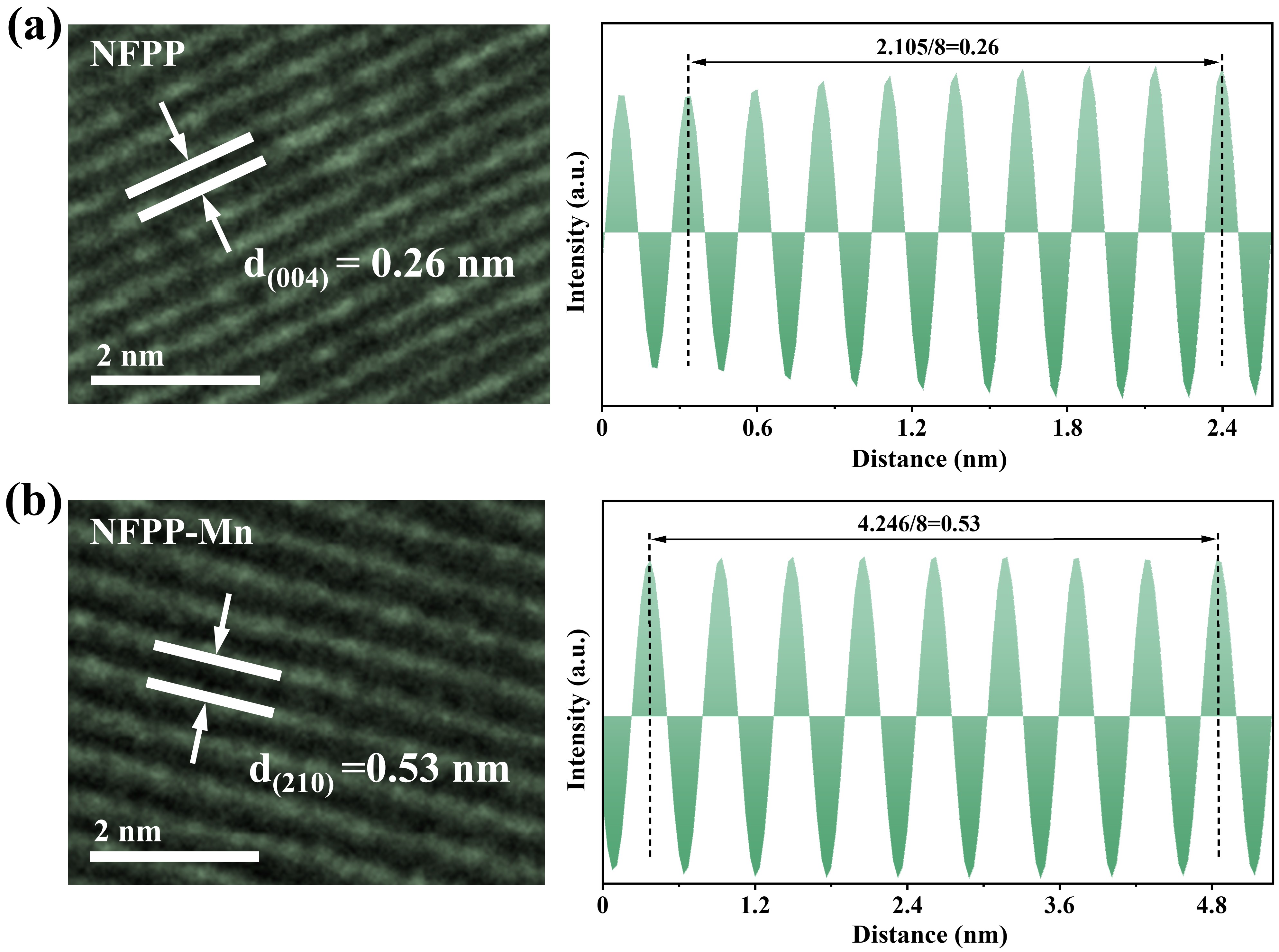


**Fig. S5** HRTEM image and the corresponding lattice spacing calculation of **a** NFPP and **b** NFPP-Mn.


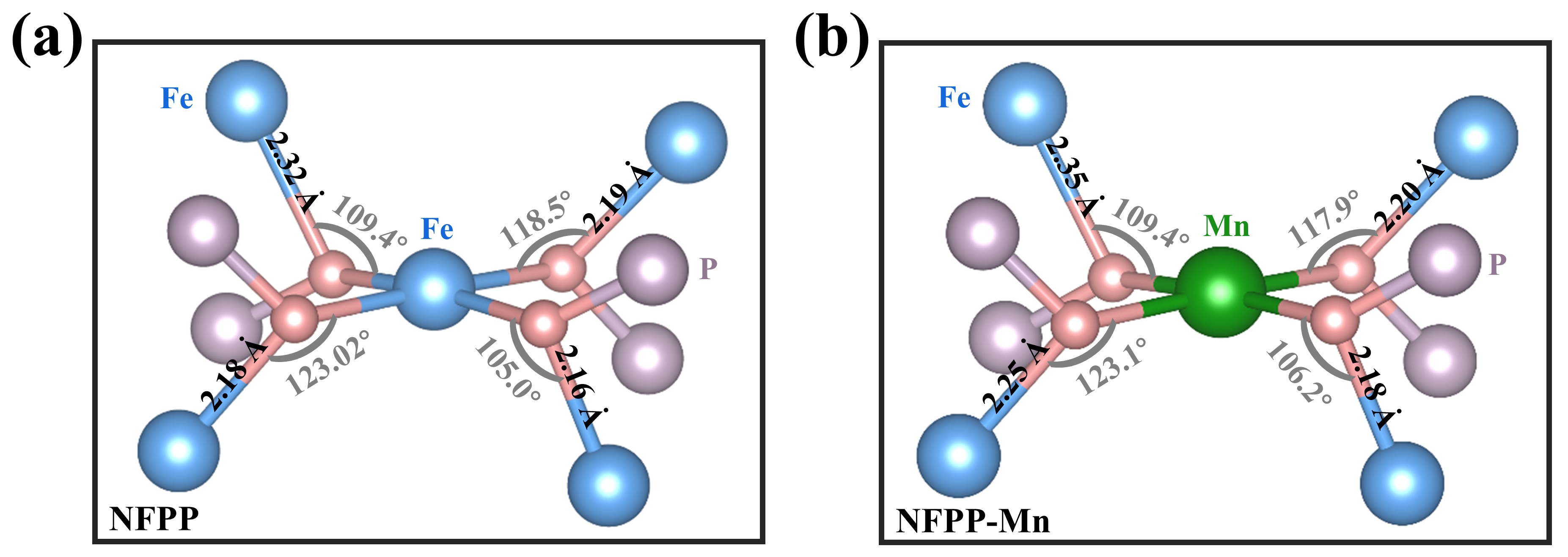


**Fig. S6** Calculated atomic structure variation of **a** NFPP and **b** NFPP-Mn material.


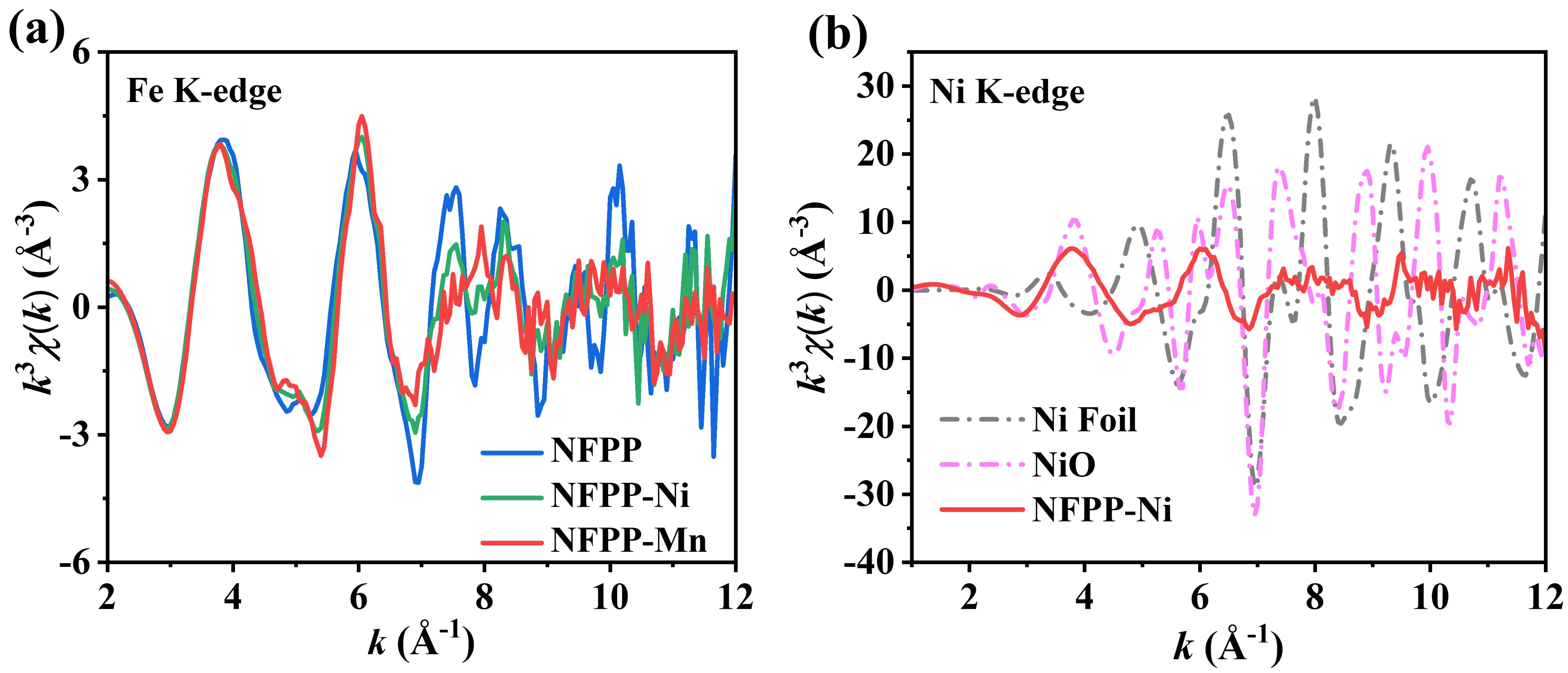


**Fig. S7** **a** Fe K-edge and **b** Ni K-edge EXAFS of oscillations.


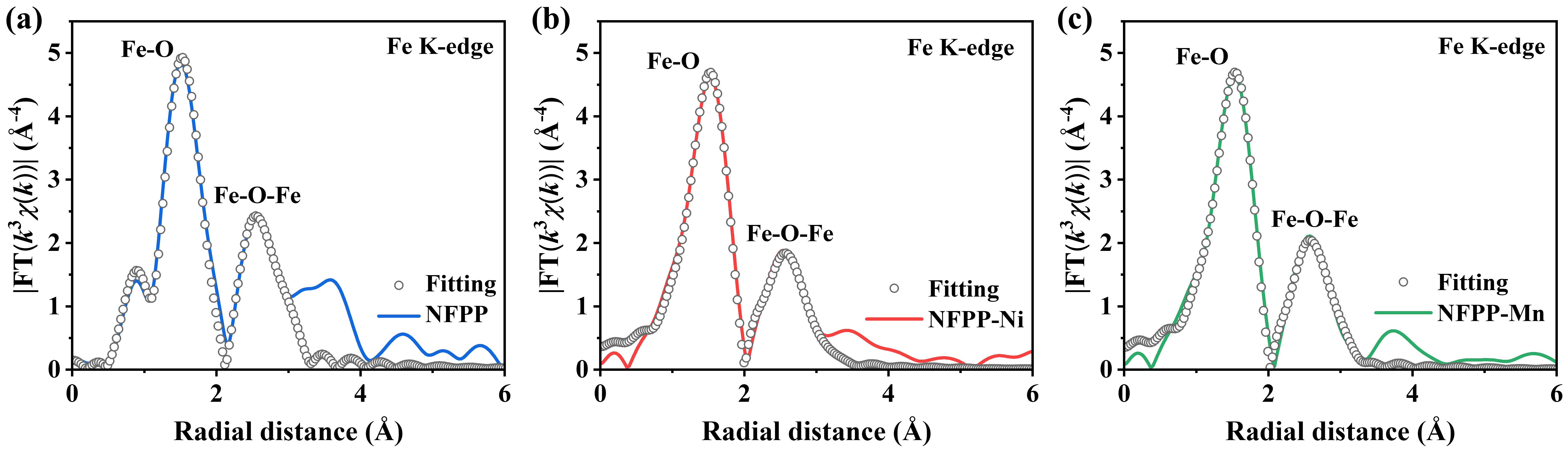


**Fig. S8** The Fourier transformed EXAFS spectra signal (lines) and fitting results (dots) of **a** NFPP, **b** NFPP-Ni and **c** NFPP-Mn samples at Fe K-edge in R spaces.


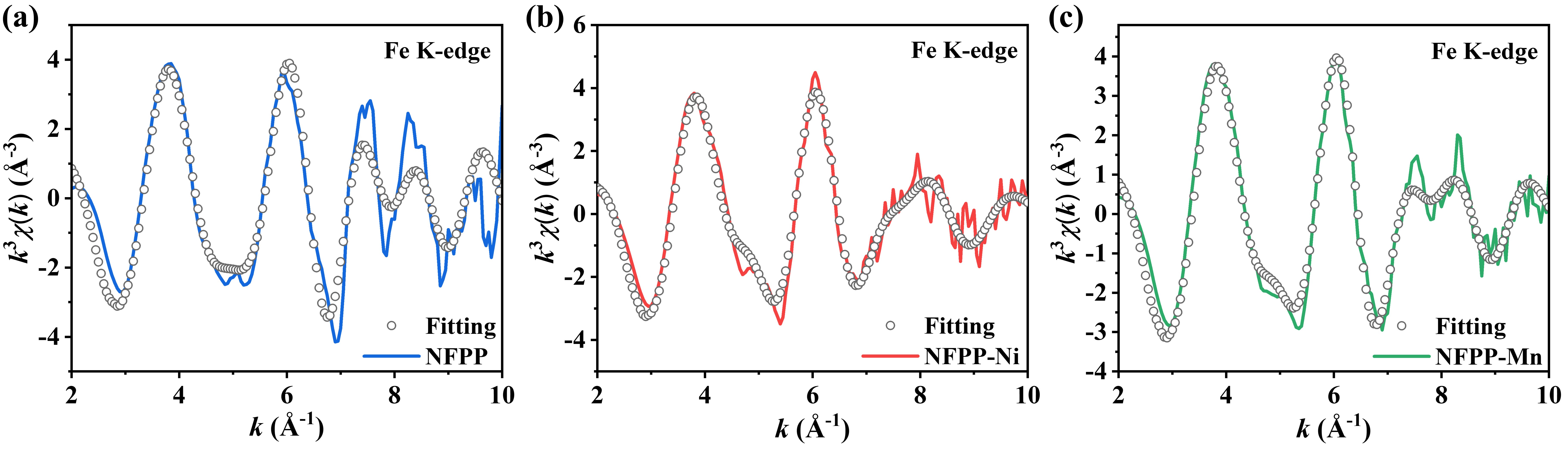


**Fig. S9** Fe K-edge EXAFS spectra signal (lines) and fitting results (dots) of **a** NFPP, **b** NFPP-Ni and **c** NFPP-Mn samples in *k* spaces.


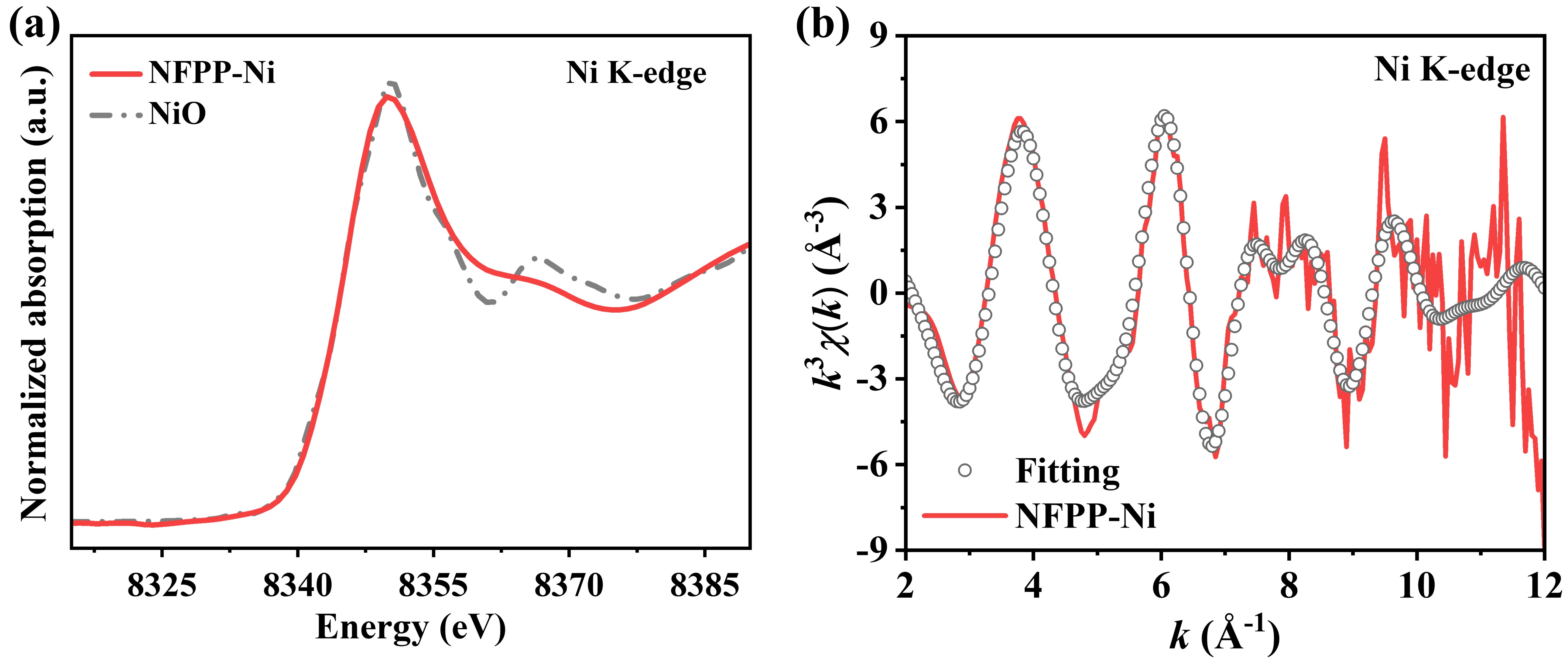


**Fig. S10** **a** The XANES spectra of NFPP-Ni at Ni K-edge. **b** Ni K-edge EXAFS spectra signal (lines) and fitting results (dots) of NFPP-Ni in *k* spaces.


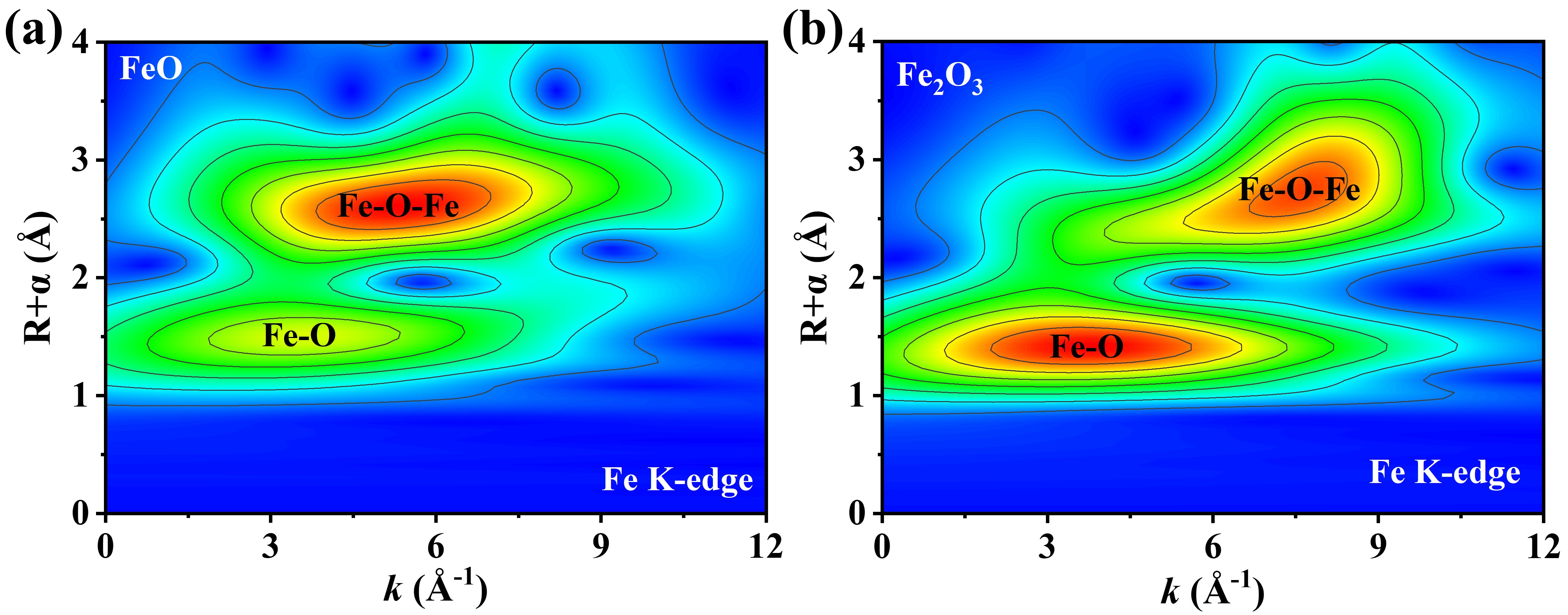


**Fig. S11** Fe K-edge WT-EXAFS spectra for **a** FeO, and **b** Fe_2_O_3_.


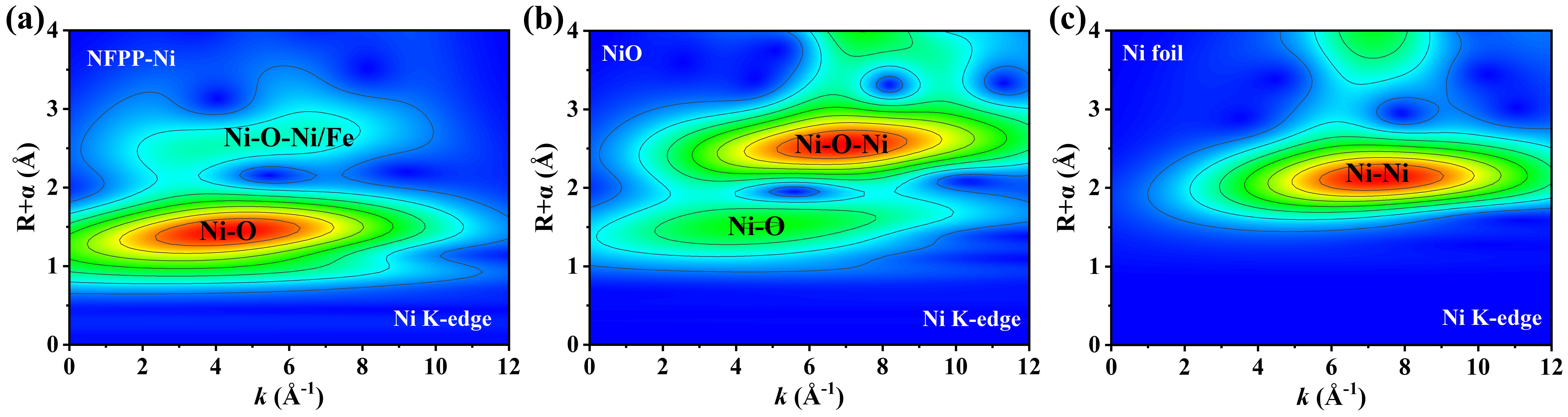


**Fig. S12** Ni K-edge WT-EXAFS spectra for **a** NFPP, **b** NiO and **c** Ni foil.


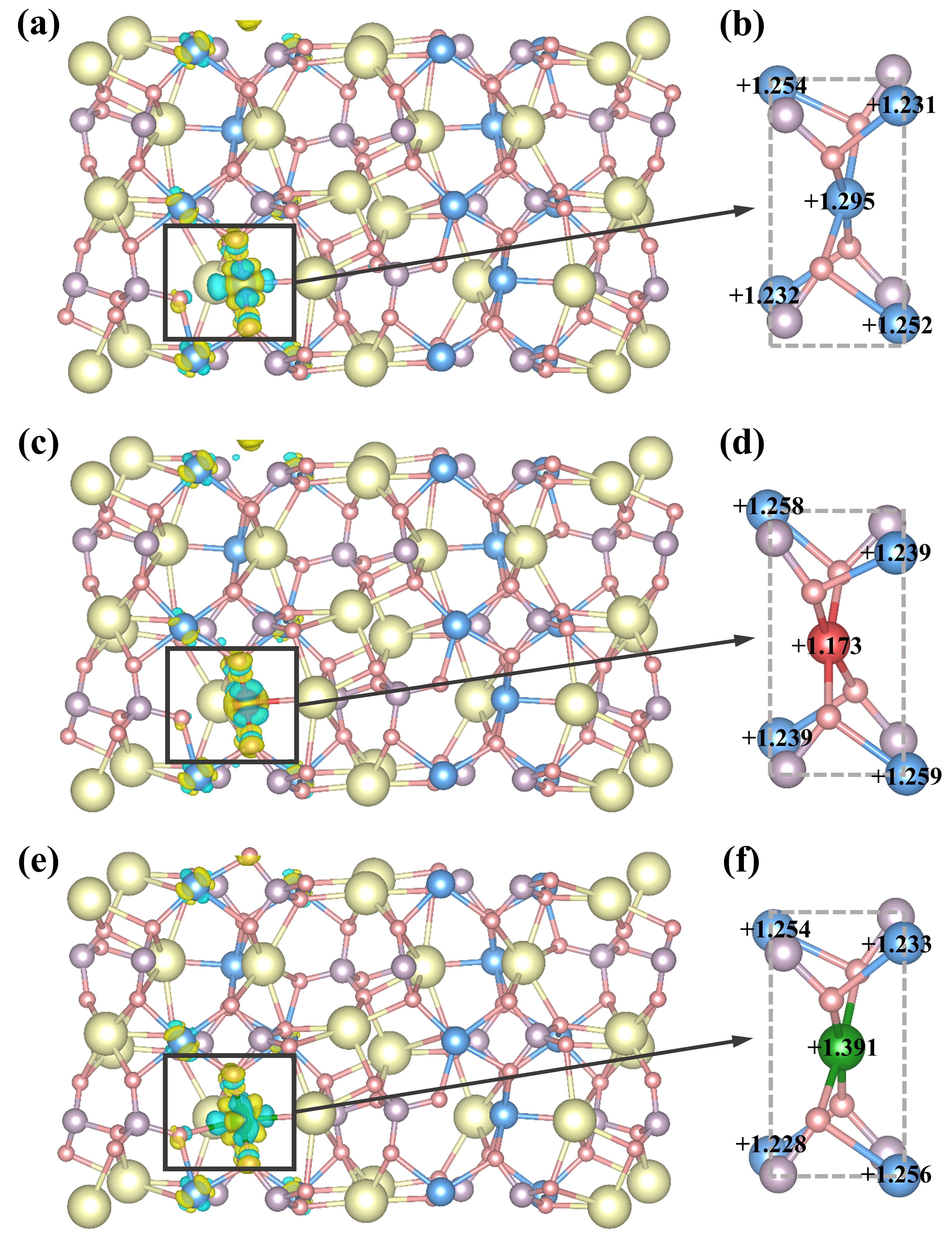


**Fig. S13** Difference charge densities and Bader charge transfers of **a, b** NFPP, **c, d** NFPP-Ni and **e, f** NFPP-Mn.


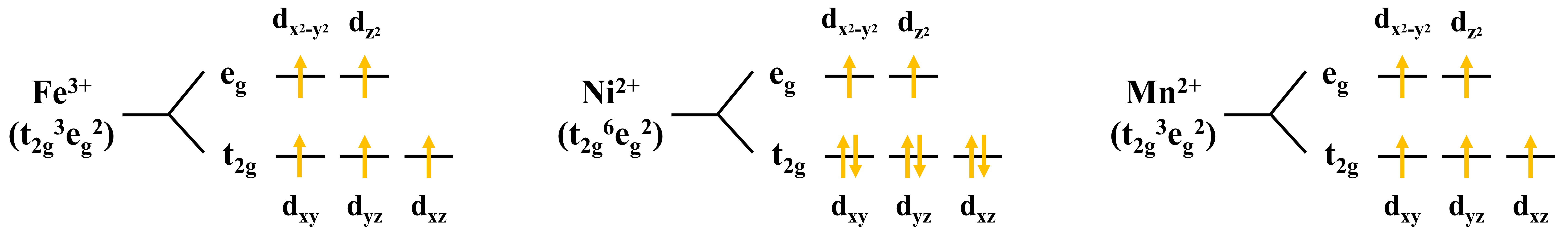


**Fig. S14** Electron configurations of 3d orbitals of Fe^3+^, Ni^2+^ and Mn^2+^.


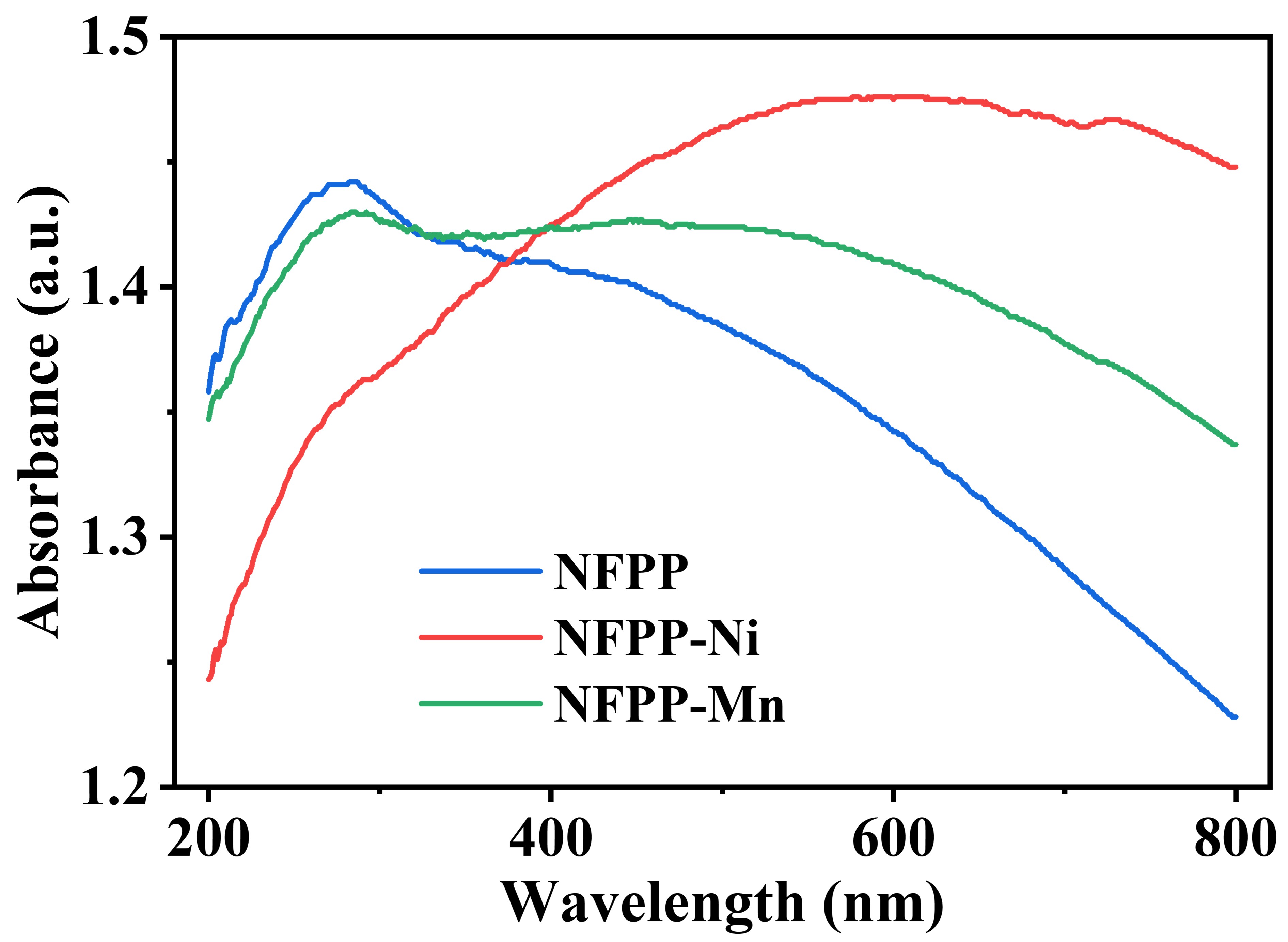


**Fig. S15** UV-vis diffuse reflectance spectra of NFPP, NFPP-Ni and NFPP-Mn samples.

The bandgap energies for all the samples were calculated using the Tauc Equation,

*(αhv)1*/*n=A(**hv-E_g_)*

where *𝛼* is the absorption coefficient, *hv* (*hv*=*hc/λ*, *c* is the speed of light, *λ* is the wavelength of light) is the incident photon energy of light, *A* is a constant, *E_g_* represents the bandgap energy, and *n* is a constant that varies depending on the nature of the electronic transition (The direct bandgap semiconductor *n* is 1/2, and the indirect bandgap semiconductor *n* is 2).^[7]^


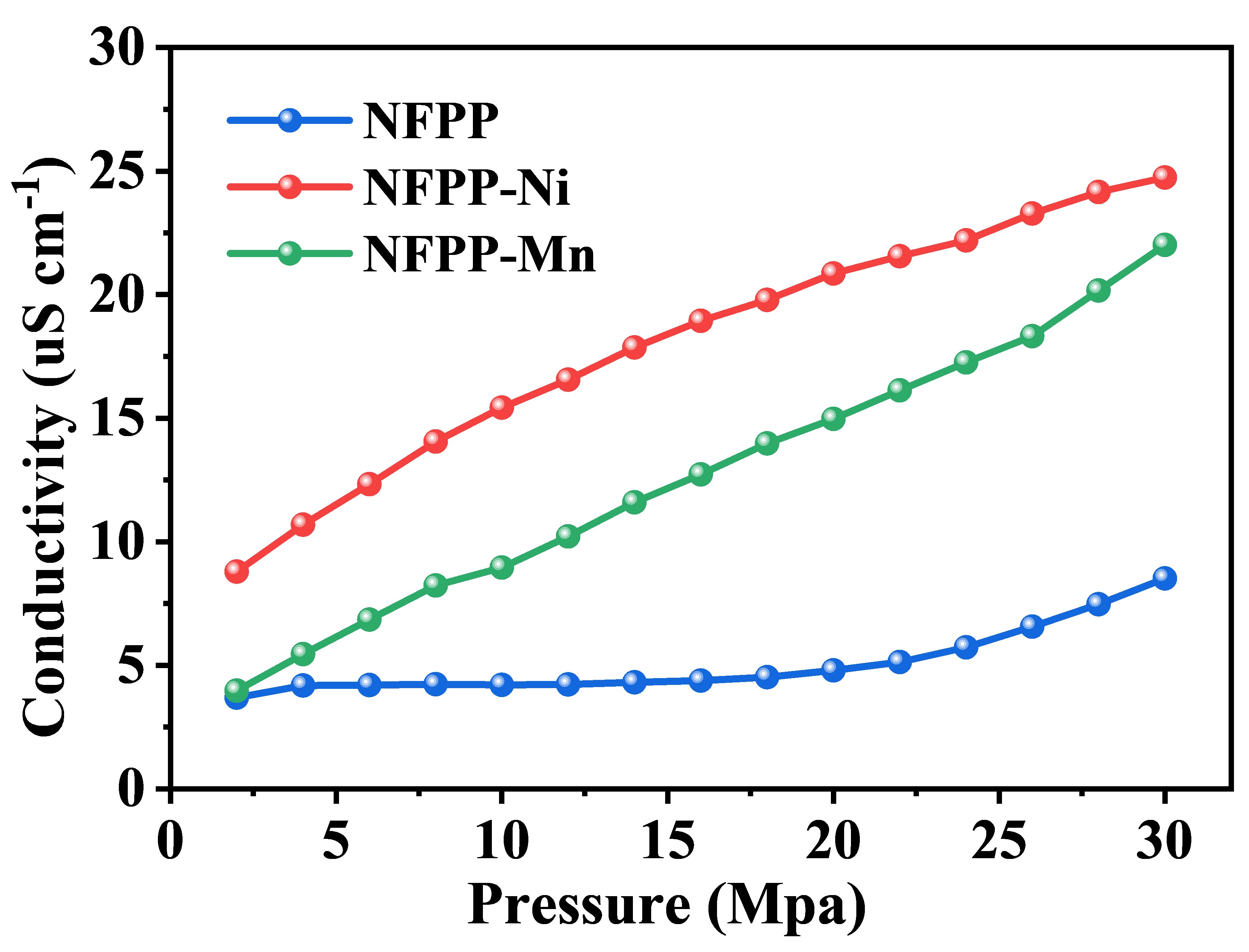


**Fig. S16** Four-terminal probe conductivity measurement of NFPP, NFPP-Ni and NFPP-Mn samples at different pressures.


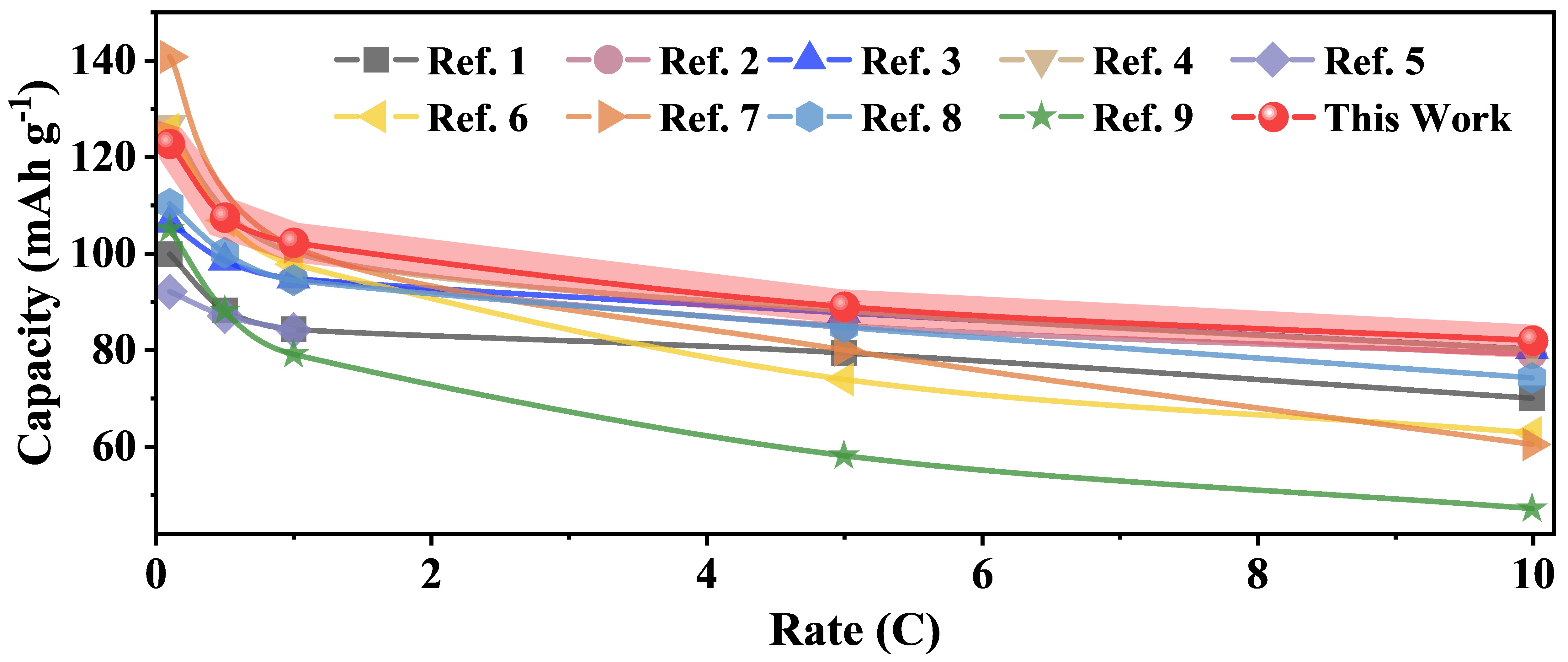


**Fig. S17** The comparison of the present study with the reported NFPP cathode for SIBs.^[7-15]^


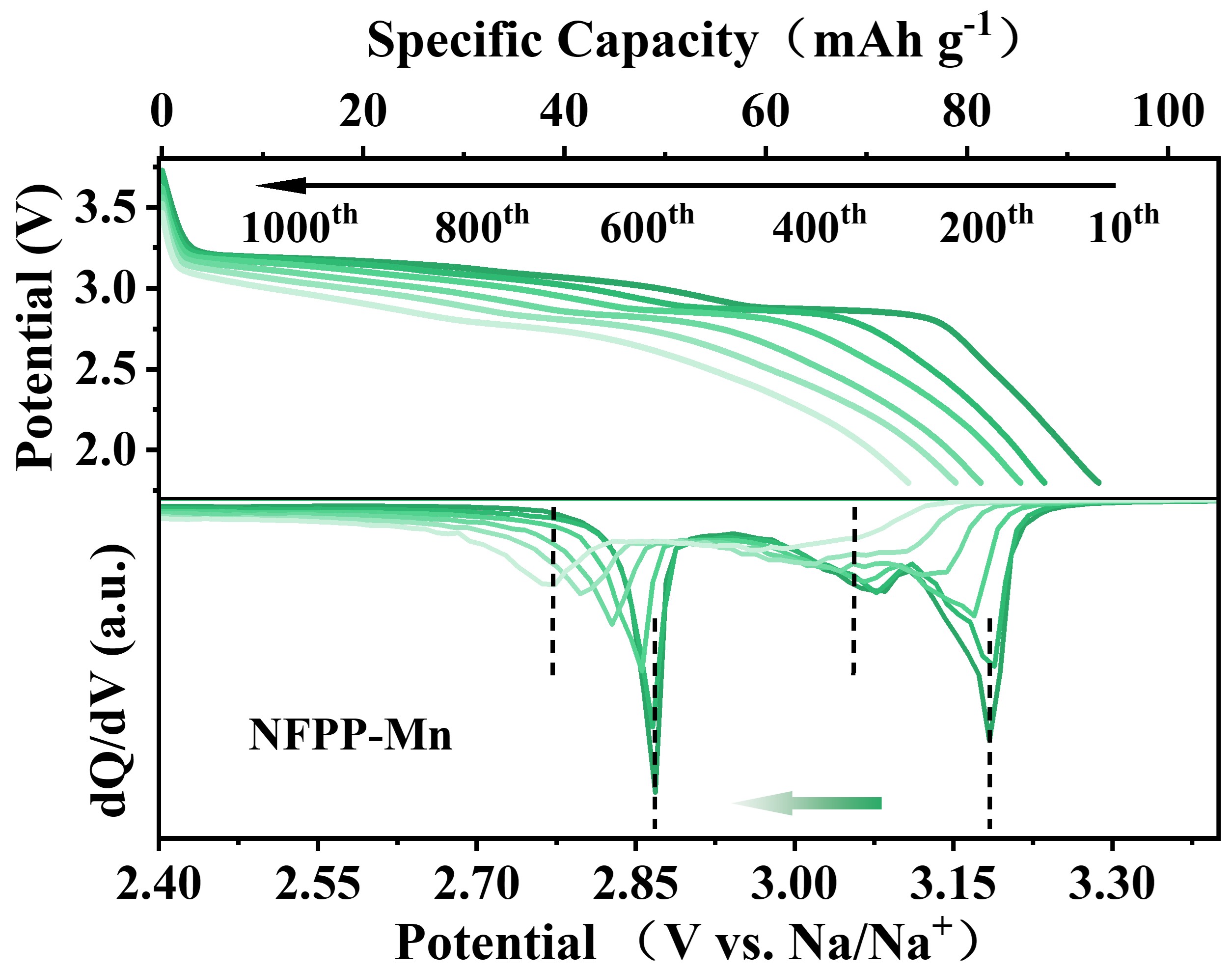


**Fig. S18** Discharge curves and corresponding dQ/dV plots of NFPP-Mn at increasing cycles from 10^th^ to 100^th^.


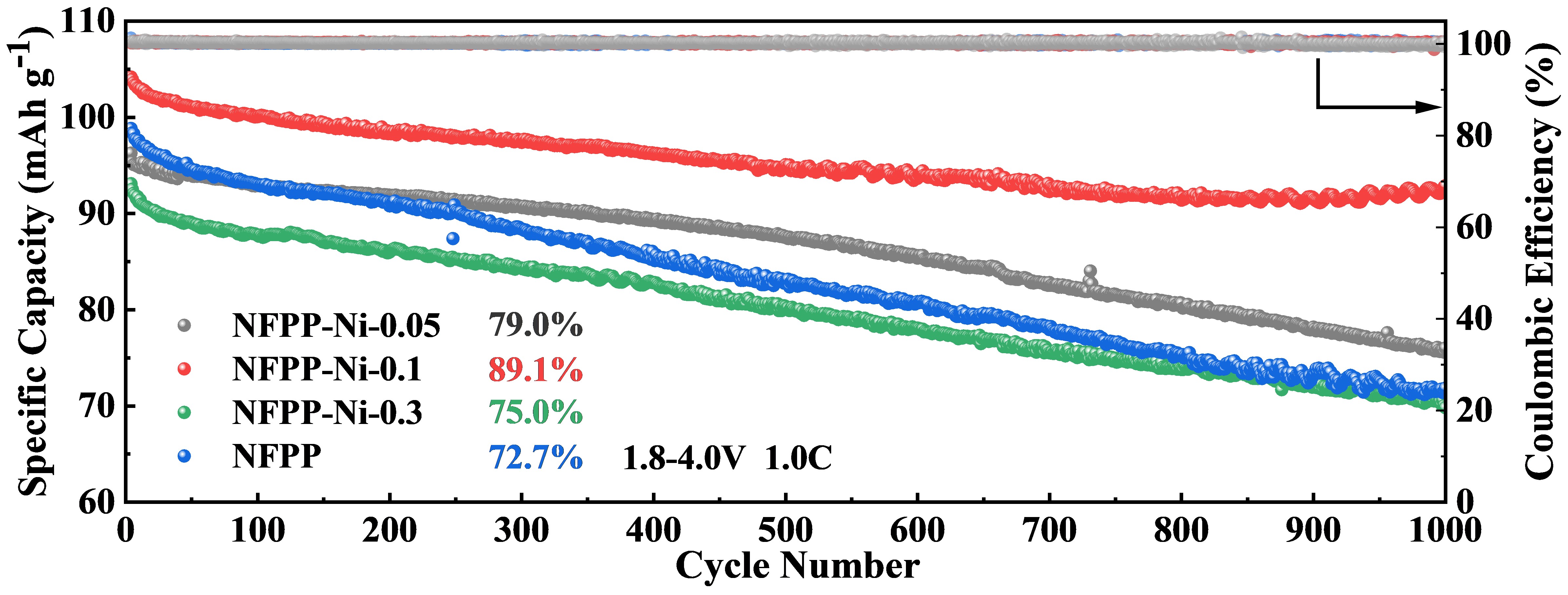


**Fig. S19** Cycling performance of NFPP, NFPP-Ni-0.05, NFPP-Ni-0.1, and NFPP-Ni-0.3 at 1C current density.


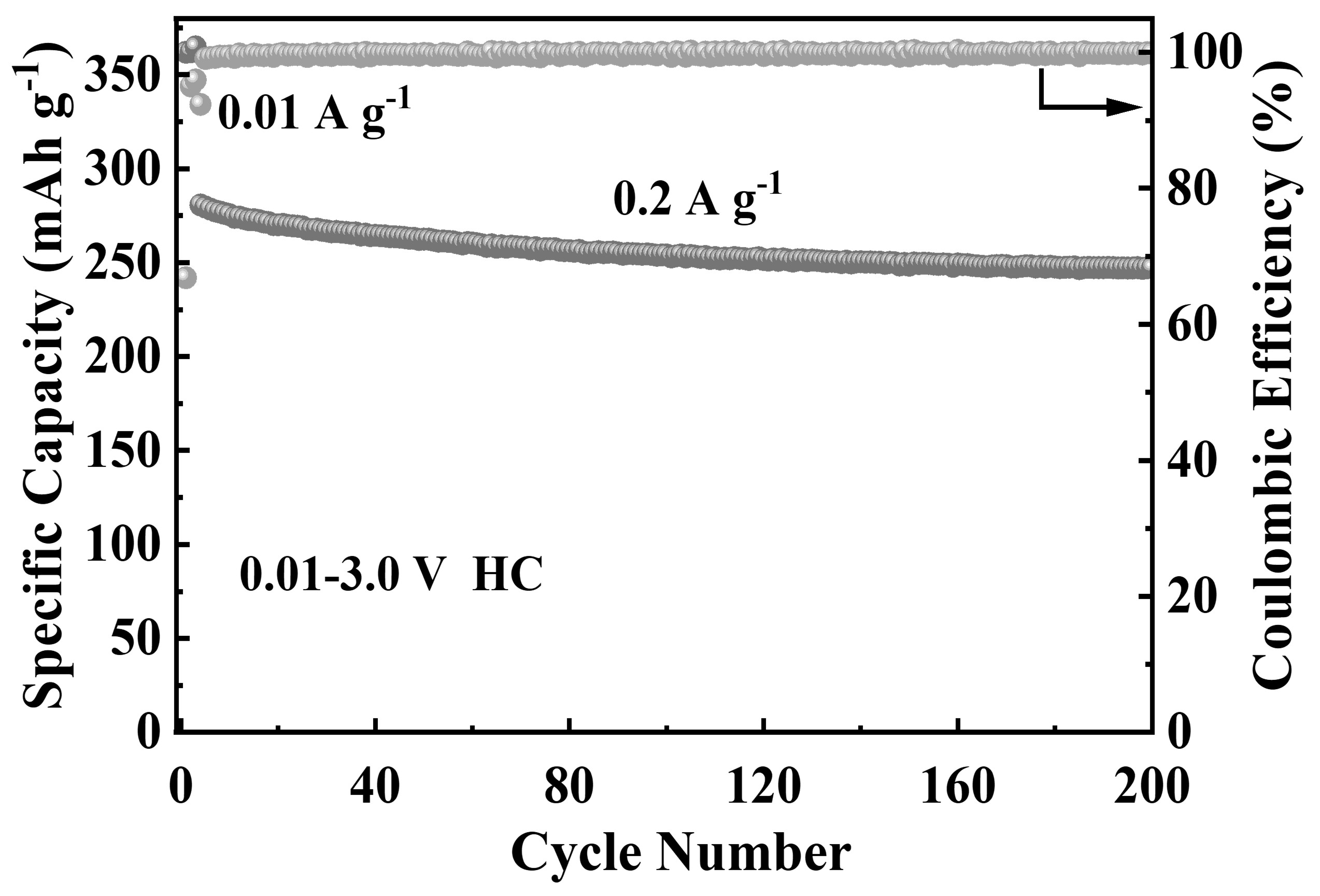


**Fig. S20** Cycling performance of hard carbon.


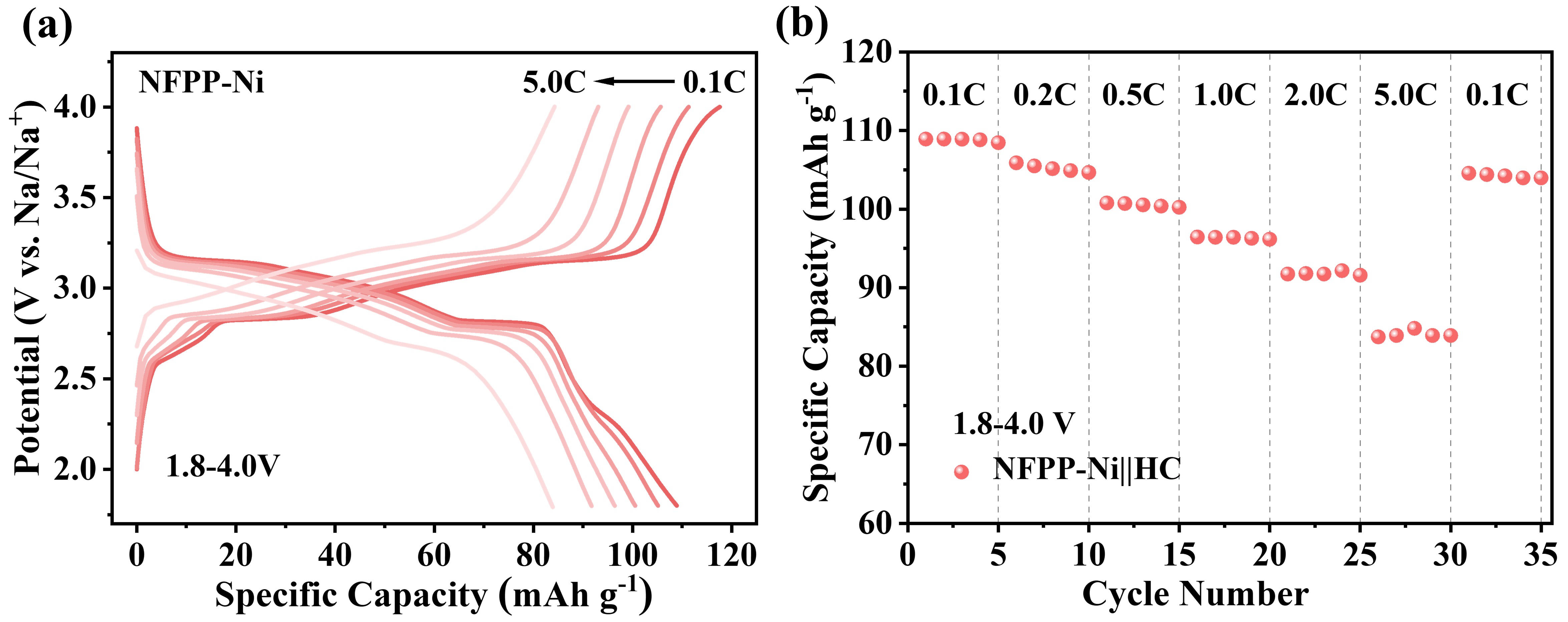


**Fig. S21** **a** The GCD profiles and **b** rate performance of NFPP-Ni ||HC full cell.


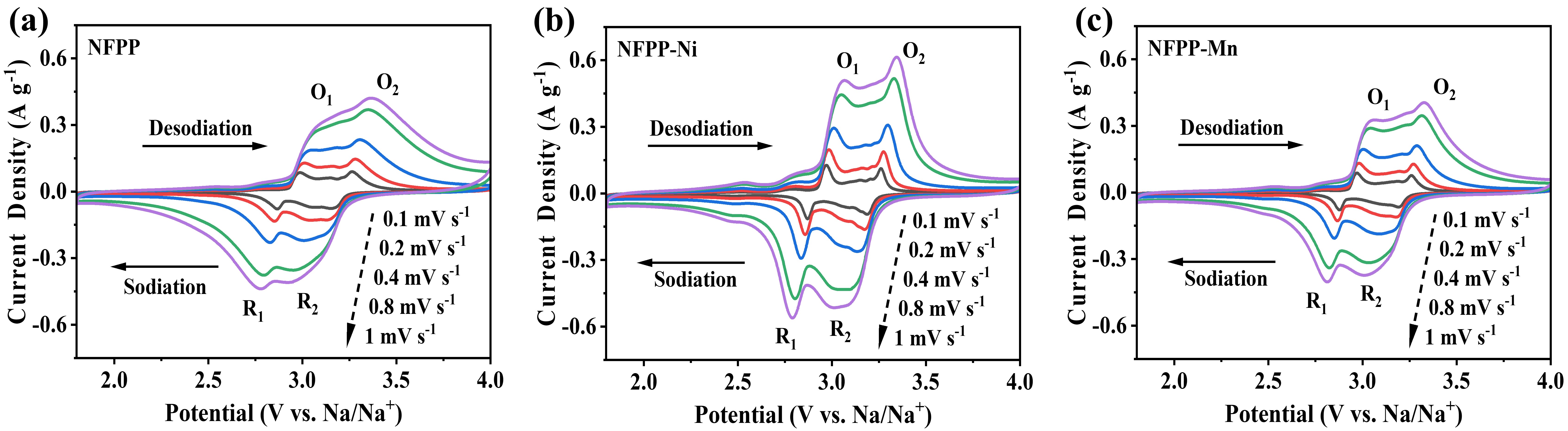


**Fig. S22** The CV curves of **a** NFPP, **b** NFPP-Ni and **c** NFPP-Mn samples at different scan rates.


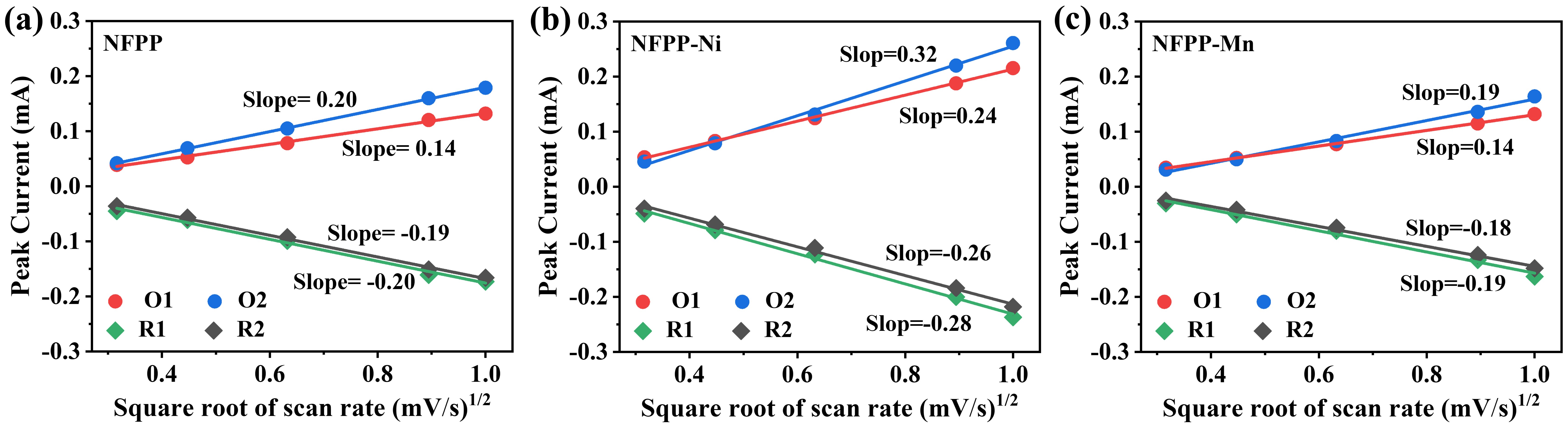


**Fig. S23** The square root of scanning rate versus peak current plots for the different peaks in the CV curves of **a** NFPP, **b** NFPP-Ni and **c** NFPP-Mn samples.

The Na^+^ diffusion coefficient in the electrode can be calculated based on the following equation:

$$i_{p}=2.69\times{10}^{5}n^{3/2}AD^{1/2}C_{0}v^{1/2}$$

Where *i_p_*, *n*, *A*, *D*, *C_0_* and *v* are the peak current (A), charge-transfer number (mol), interfacial contact area between electrolyte and electrode (cm^2^), Na^+^ diffusion coefficient (cm^2^ s^-1^), Na^+^ bulk concentration in electrode (mol L^-1^), and scan rate (mv s^-1^), respectively.


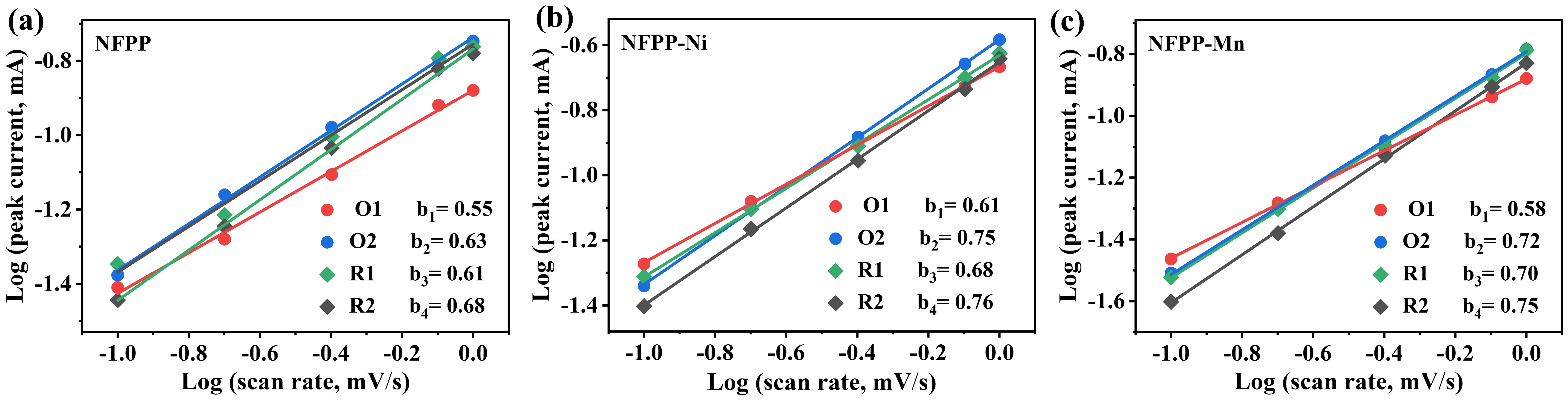


**Fig. S24** The log (*i*) versus log (*v*) plots for the different peaks in the CV curves of **a** NFPP, **b** NFPP-Ni and **c** NFPP-Mn samples, where i is the peak current and *v* is the scan rate.

The charge-storage mechanism is analyzed based on the following equations:

$$i_{p}=av^{b}$$

$$\log\left( i_{p} \right)=b\times\log\left( v \right)+loga$$

Where *i*, *v*, *a*, and *b* are peak current (A), scan rate (mv s^-1^), and adjustable parameters. If the b-value approaches 1.0, the electrochemical reaction system is mainly controlled via surface-controlled pseudocapacitive behavior (capacitive response), while if the b-value is close to 0.5, the insertion/de-insertion process (Faradaic intercalation) for Na^+^ dominates the system (diffusion limitation response).


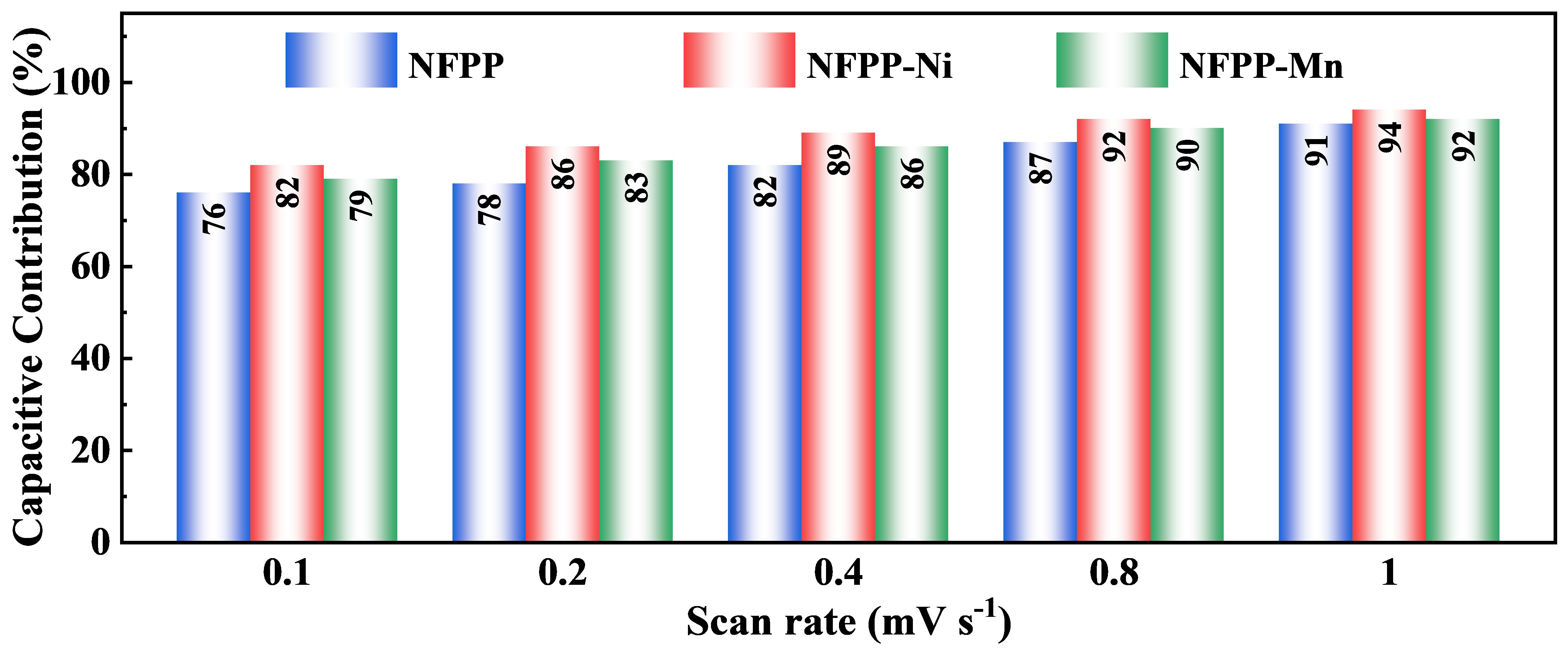


**Fig. S25** Capacitive contribution ratios of NFPP, NFPP-Ni and NFPP-Mn samples at various scan rates.

The pseudocapacitive contribution can be calculated according to the following equation:

$$\frac{i(V)}{v^{\frac{1}{2}}}=k_{1}v^{\frac{1}{2}}+k_{2}$$

where *k*_1_*v* and *k*_2_*v*^1/2^ represent the capacitive and diffusion-limited currents, respectively.


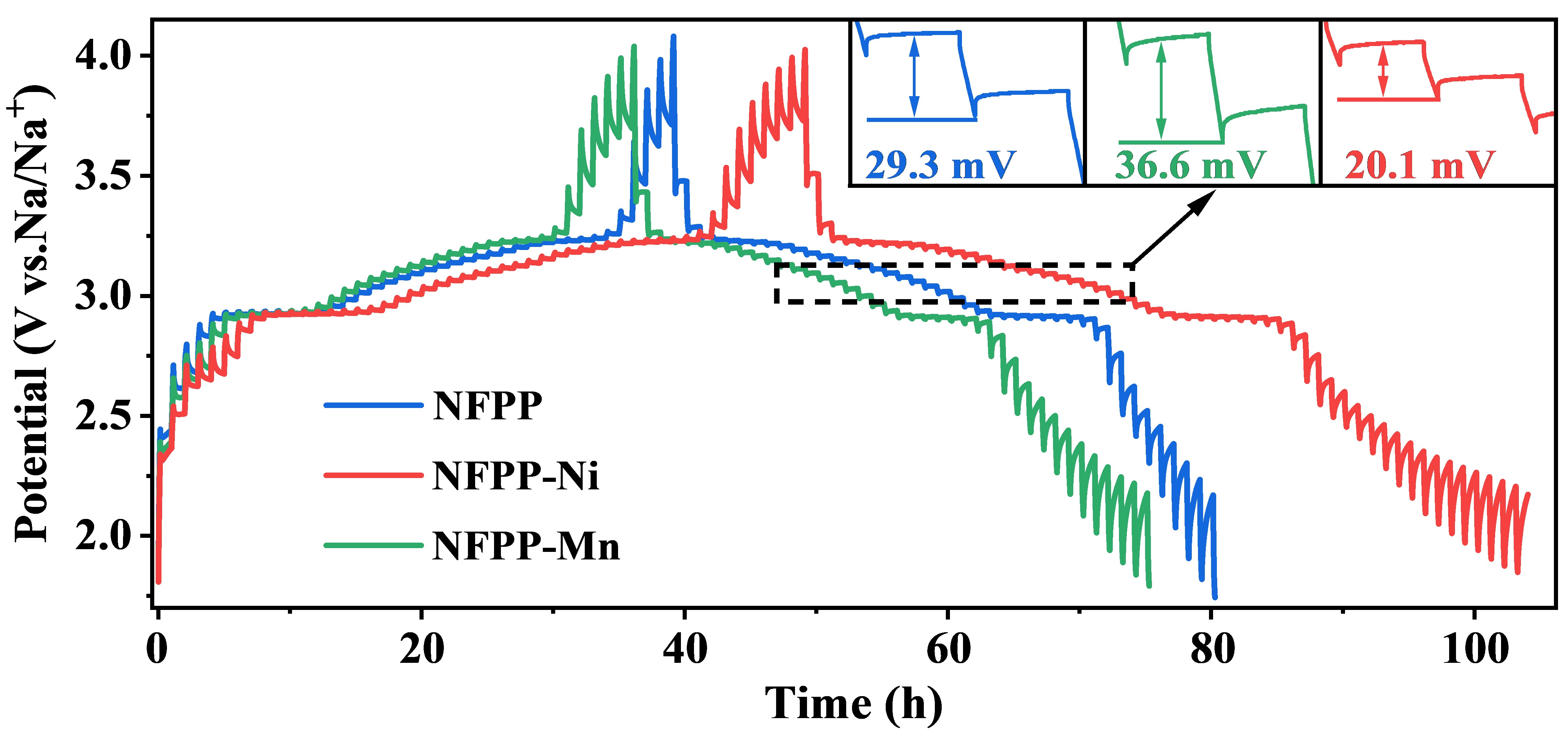


**Fig. S26** Galvanostatic intermittent titration technique (GITT) profile of NFPP, NFPP-Ni and NFPP-Mn sample.

The sodium-ions diffusion coefficients were measured by Galvanostatic Intermittent Titration Technique (GITT). To ensure complete CEI formation, each coin cell was cycled three times at 0.1C before conducting the GITT experiments. Subsequently, the cell was charged and discharged at 0.1C for 20 min, and then relaxed for 240 min to allow the voltage reach equilibrium. The coefficient of diffusion D_Na_^+^ can be calculated based on the following equation:

$D_{\mathrm{Na}}=\frac{4}{\pi\tau}{(\frac{m_{B}V_{B}}{M_{B}S})}^{2}{(\frac{{\Delta E}_{s}}{{\Delta E}_{\tau}})}^{2}$ (*τ « L*^2^/*D*)

In the above equation, *τ* is the constant current pulse time (600 s), *m_B_*, *V_B_*, *S*, and *M_B_* are the mass of the active material, molar volume (determined by XRD), electrode-electrolyte interface area, and molecular weight, respectively. *ΔE_s_* is the difference between two consecutive stable voltages after relaxation, and *ΔE_τ_* is the total change of cell voltage during a constant current pulse excluding the IR drop; *L* is the thickness of electrode.


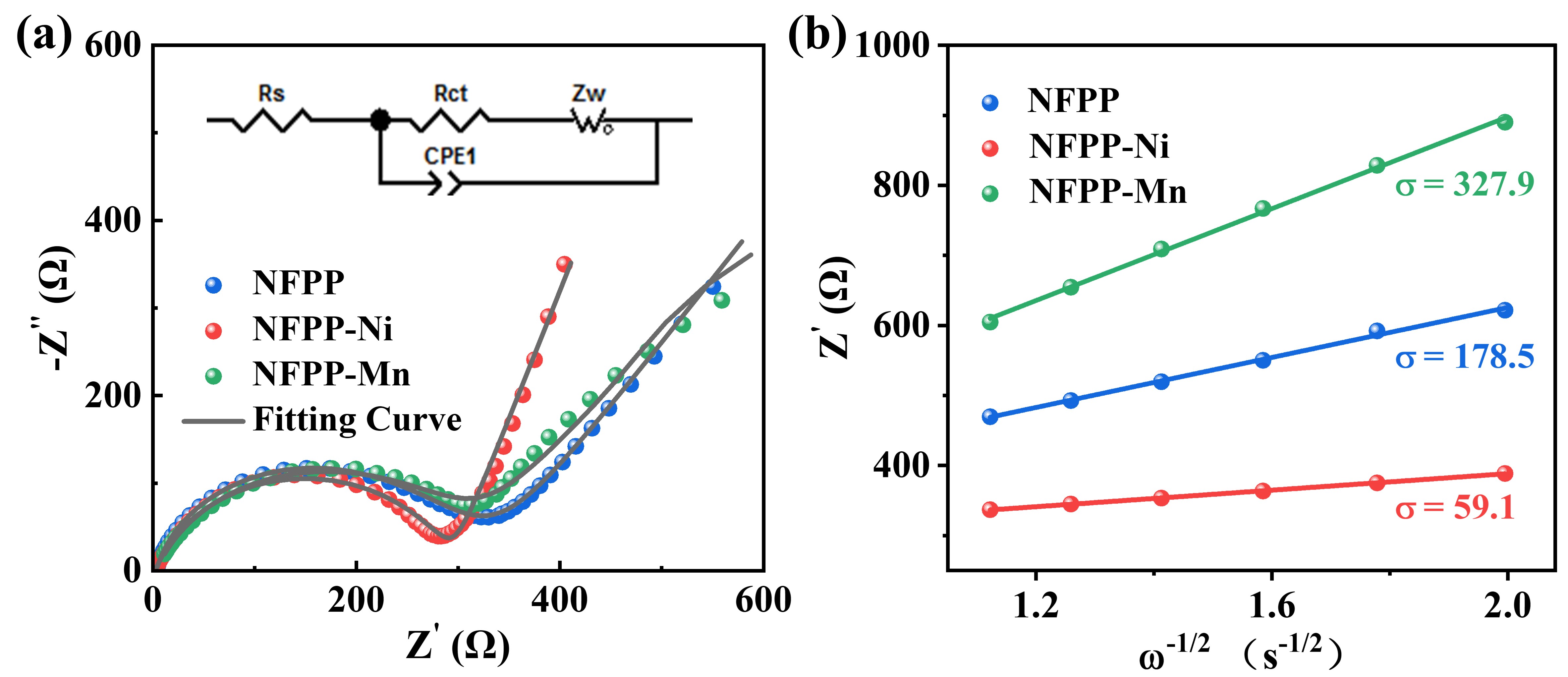


**Fig. S27** EIS spectra of NFPP, NFPP-Ni and NFPP-Mn samples. **a** Nyquist plot, **b** variations and fittings between Z’ versus ω^-1/2^ plot.

The Nyquist plots of NFPP, NFPP-Ni and NFPP-Mn samples, including a semicircle in the high frequency region, which refers to the interfacial charge transfer resistance (R_ct_) between electrode and electrolyte, and a sloping line in the low frequency region, which represents the Warburg impedance (*Z_w_*), associating with Na+ ion diffusion in the bulk material.

The calculation for *D*_Na_^+^ can be written as:

$$D_{{Na}^{+}}=\frac{R^{2}T^{2}}{2A^{2}n^{4}F^{4}C^{2}\sigma^{2}}$$

$$Z^{'}=R_{e}+R_{ct}+{\sigma\omega}^{-1/2}$$

Where *D*_Na_^+^, *R*, *T*, *A*, *n*, *F*, *C*, and *σ* is diffusion coefficient, gas constant, room temperature, cathode material area (*A* = 4.52 cm^2^), number of the insertion/extraction ions during charge/discharge, Faraday constant, sodium ion concentration in active material, and Warburg factor, respectively.


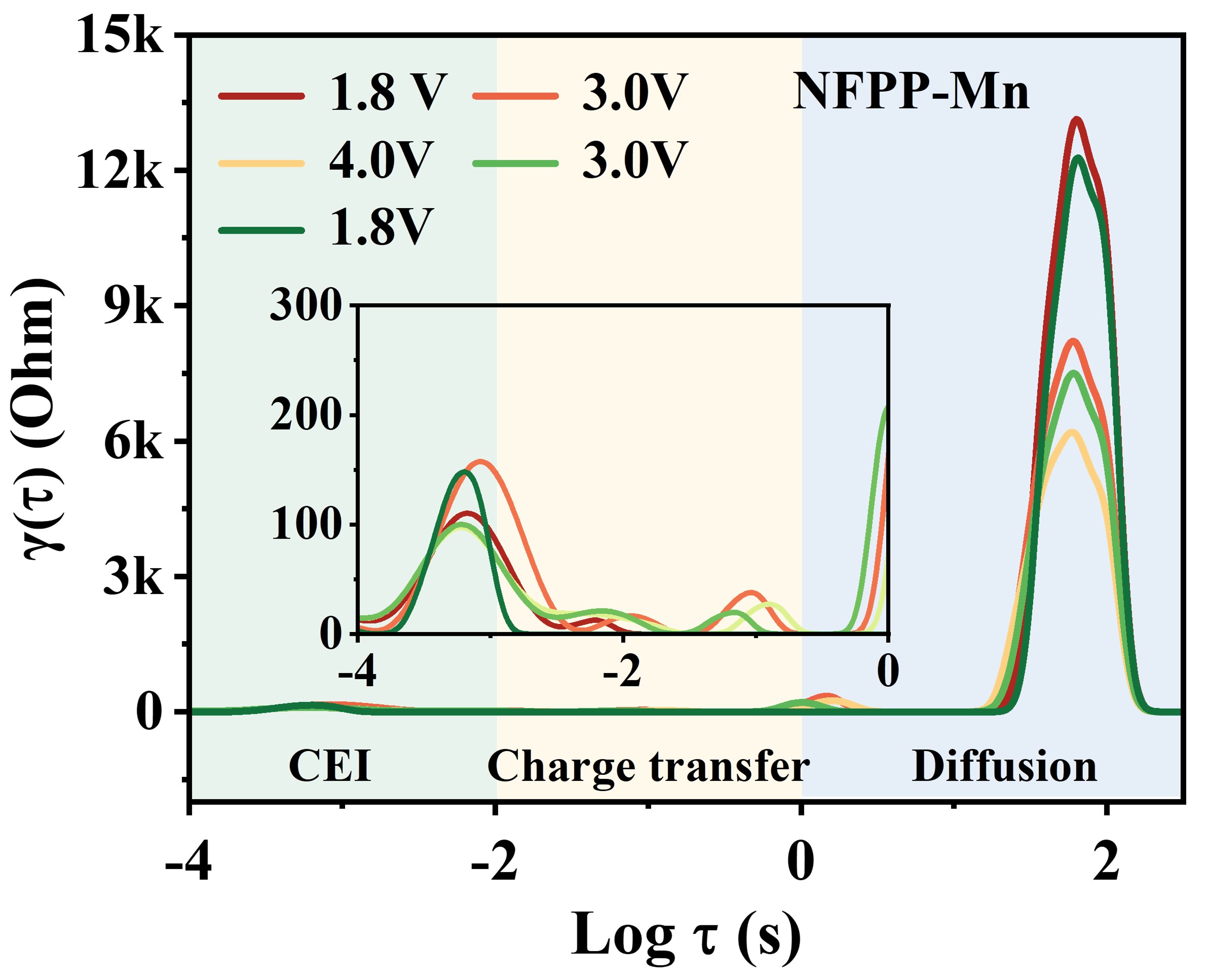


**Fig. S28** DRT analysis of EIS test of NFPP-Mn sample.


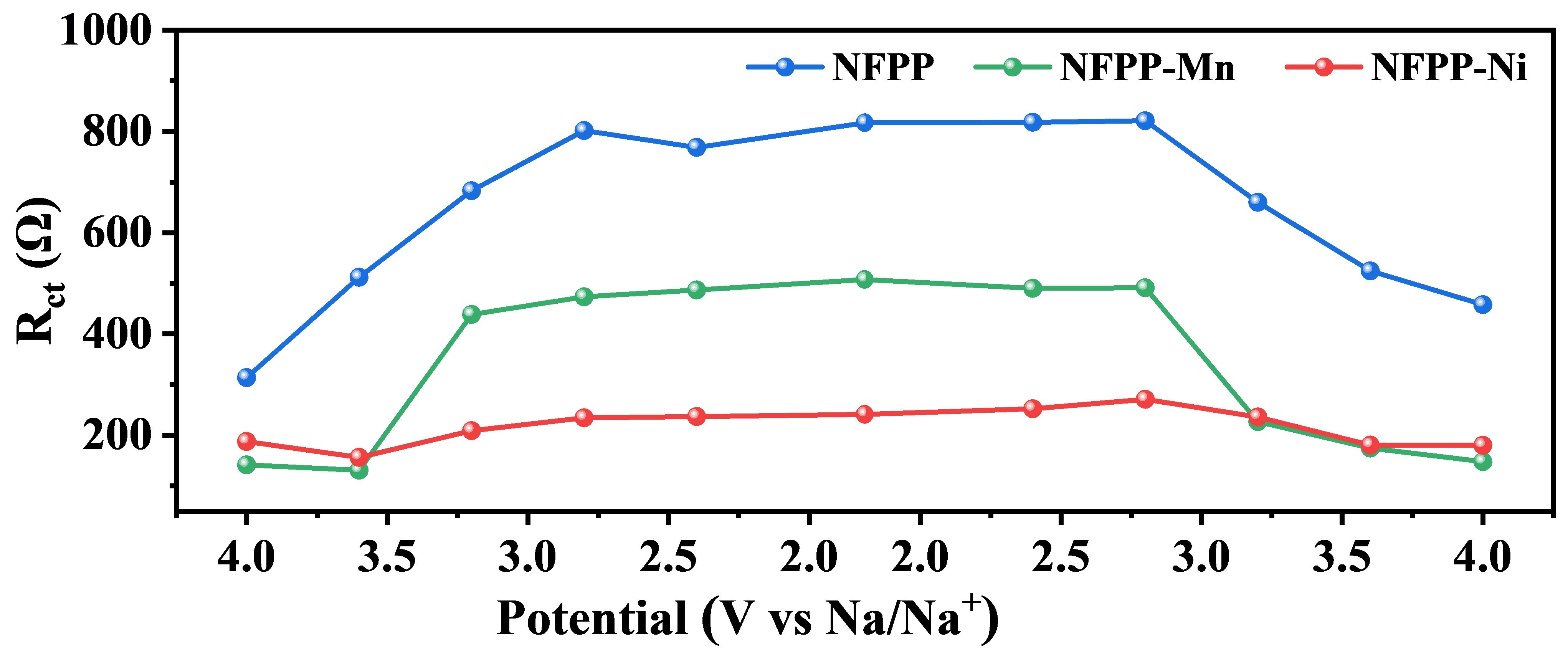


**Fig. S29** The corresponding changes of R_ct_ vs. potential.


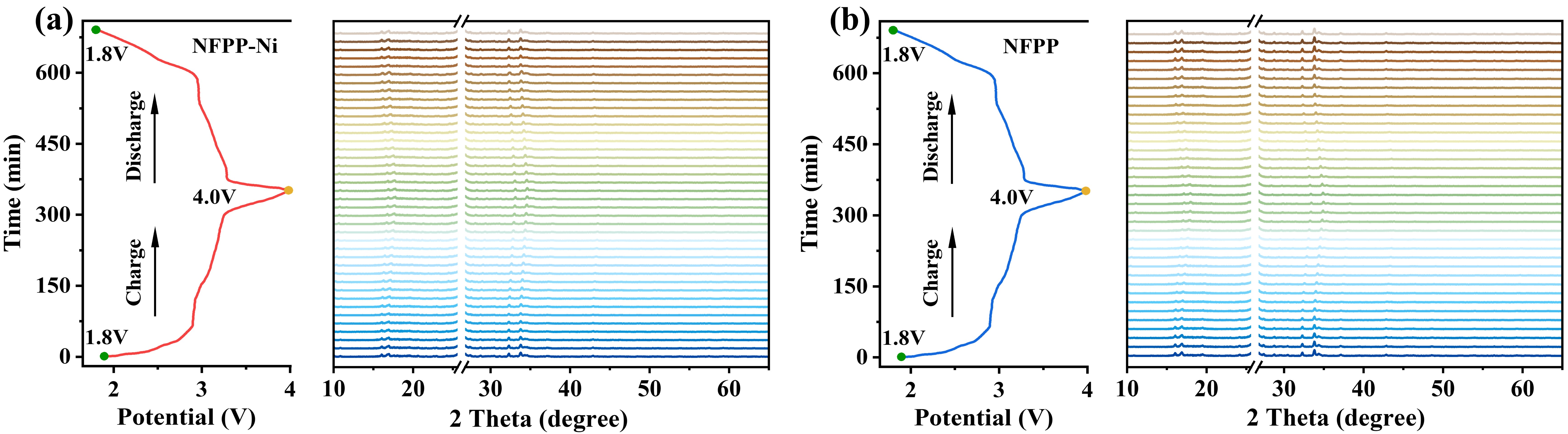


**Fig. S30** The corresponding in-situ XRD pattern curves at various charge/discharge states and the charge/discharge curves of **a** NFPP-Ni and **b** NFPP at 0.1C between 1.8 and 4.0 V in SIB.


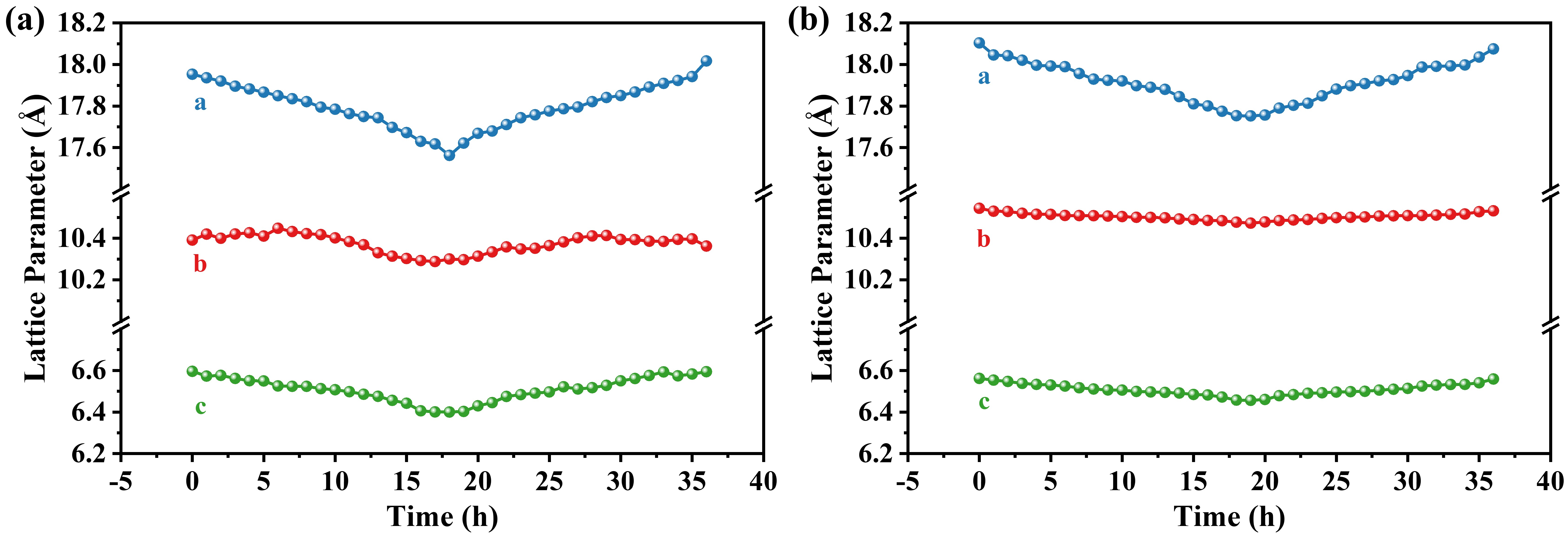


**Fig. S31** The calculated evolution of lattice parameters a, b, and c during in-situ charge and discharge processes for (a) NFPP, and (b) NFPP-Ni.


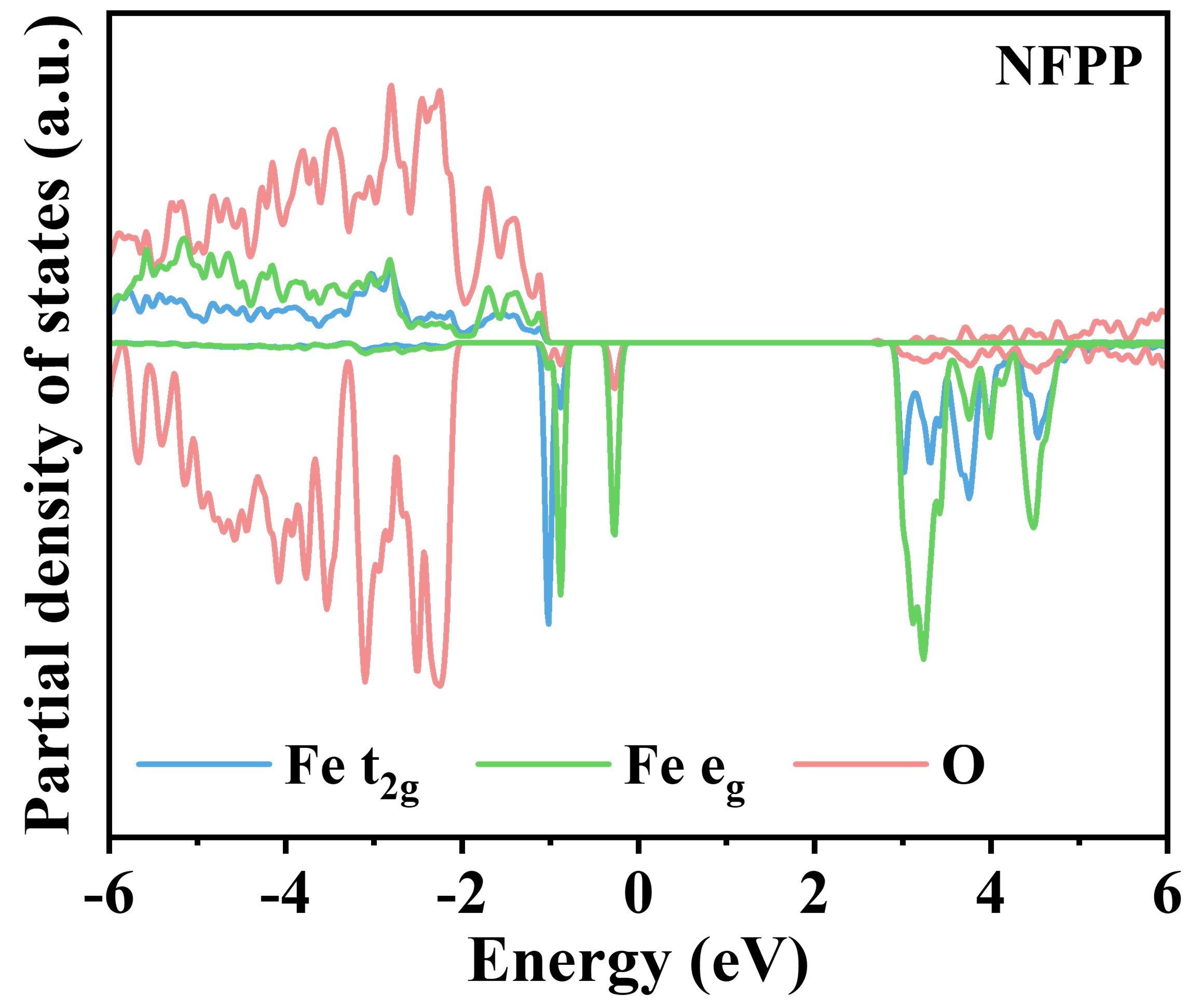


**Fig. S32** The PDOS of Fe e_g_, Fe t_2g_ and O for NFPP.


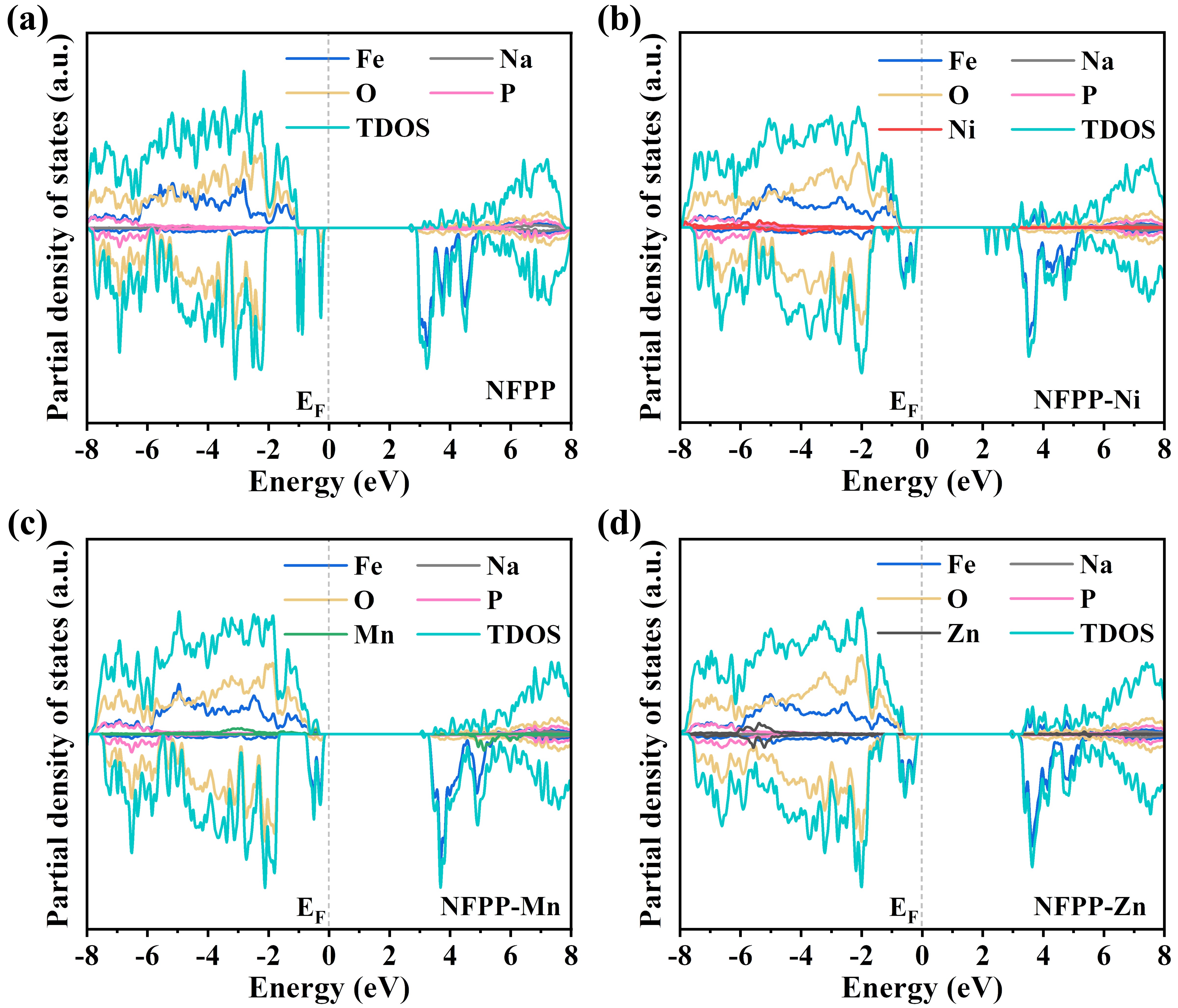


**Fig. S33** The PDOS of **a** NFPP, **b** NFPP-Ni, **c** NFPP-Mn and **d** NFPP-Zn sample.


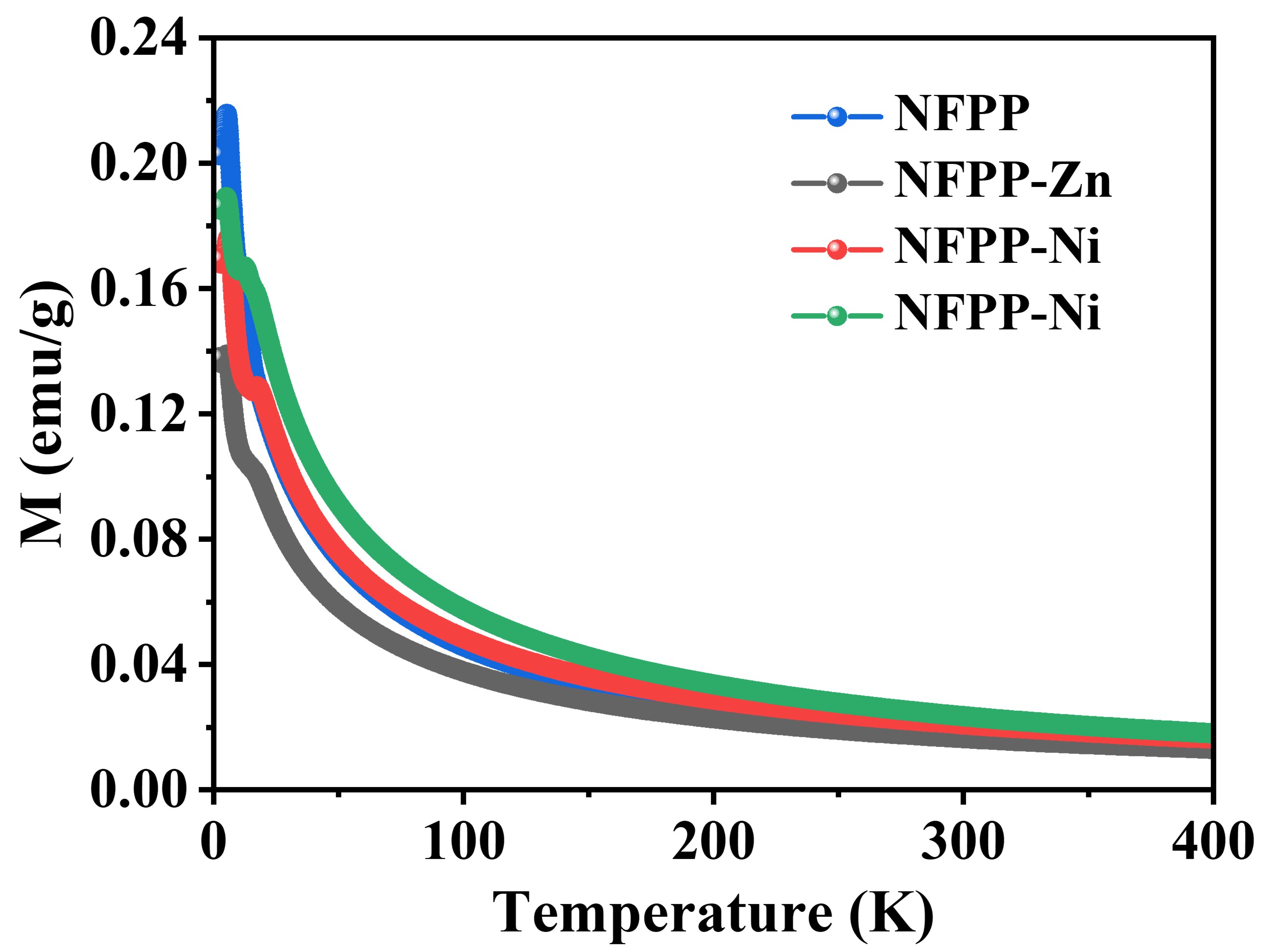


**Fig. S34** Temperature dependent magnetization under H = 0.8 kOe of NFPP-Zn, NFPP, NFPP-Ni and NFPP-Mn samples.

The effective magnetic moments (*μ_eff_*) for all the samples are obtained by $\text{μ}\text{ = }\sqrt{\text{8C}}\text{μ}_{\text{B}}$, where C is Curie constant and obtained from the fittings of the susceptibility (χ = M/H) above 150 K by a Curie-Weiss law. Using these values, the volume fractions of metal ions in HS and LS states can be calculated from the relationship: $\text{μ}_{\text{eff}}\text{ = }\text{g}\text{μ}_{\text{B}}\sqrt{\text{S}_{\text{HS}}\left( \text{S}_{\text{HS}}\text{+1} \right)\text{V}_{\text{HS}}\text{+}\text{S}_{\text{LS}}\left( \text{S}_{\text{LS}}\text{+1} \right)\text{V}_{\text{LS}}}$, where *S_HS_* (= 2) and S_LS_ (= 0) are the S values, and *V_HS_* and *V_LS_* (= 1 – *V_HS_*) are the volume fractions for metal ions in HS and LS states, respectively. Consequently, the *e_g_* electron (χ) can be further calculated by χ = *S_HS_* × *V_HS_* + *S_LS_* × *V_LS_* = 2 *V_HS_*.


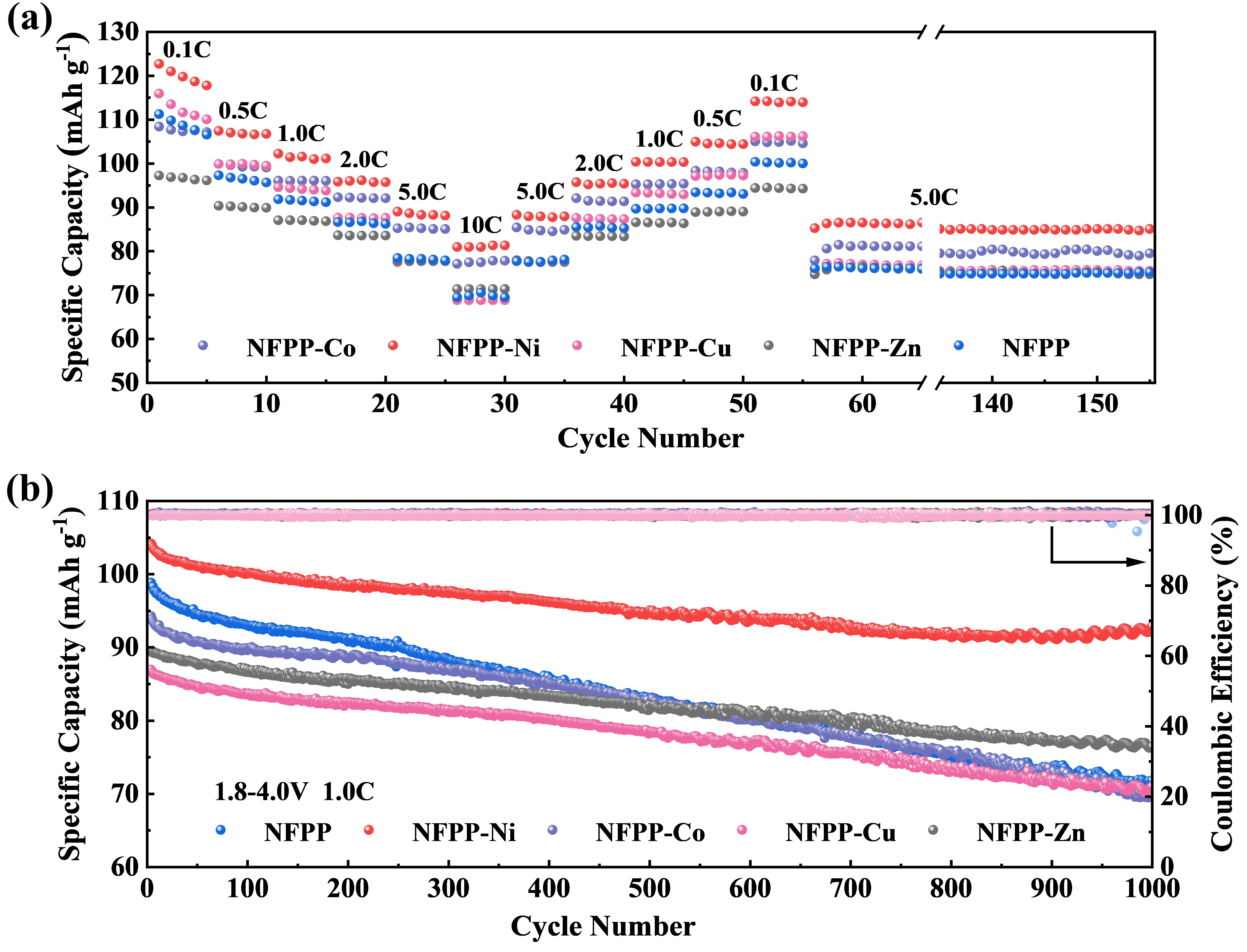


**Fig. S35** **a** Rate and **b** cycle performance of NFPP, NFPP-Ni, NFPP-Co, NFPP-Cu and NFPP-Zn.


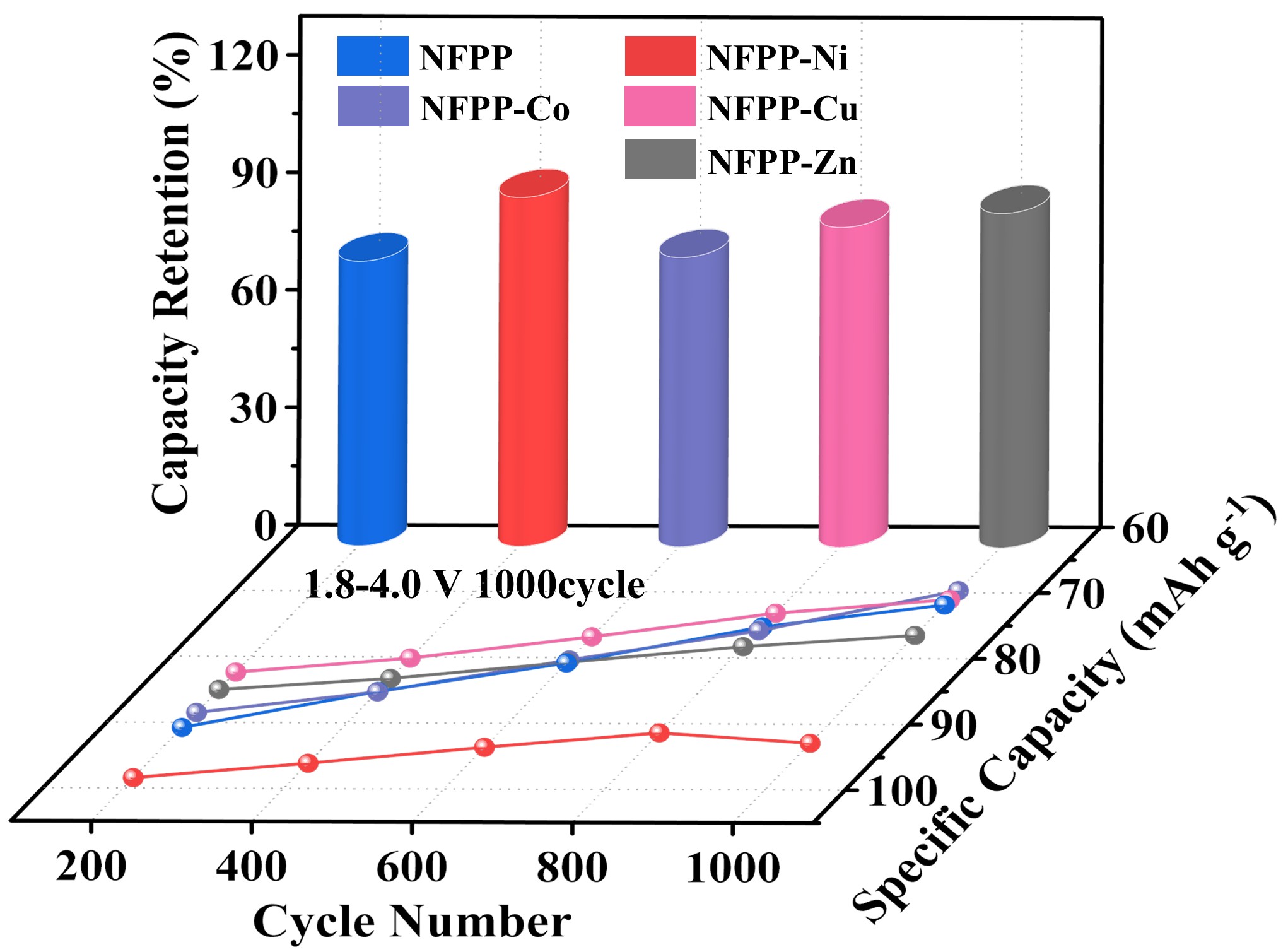


**Fig. S35** The cycling performance.

**Table S1.** ICP-MS analysis of NFPP, NFPP-Ni and NFPP-Mn samples.

| Sample | Content (mol %) | | | Na : Fe |
| --- | --- | --- | --- | --- |
|  | Fe | Na | |  |
| NFPP | 0.4125 | 0.5942 | | 1.44 |
| Sample | Content (mol %) | | | |
|  | Fe | Ni | Mn | M : Fe |
| NFPP | 45.35 | / | / | 0 : 1 |
| NFPP-Ni | 43.87 | 1.65 | / | 0.0376 : 1 |
| NFPP-Mn | 44.64 | / | 1.69 | 0.0378 : 1 |

**Table S2.** Detailed structural information of NFPP, NFPP-Ni and NFPP-Mn after Rietveld determined.

| Sample | NFPP | NFPP-Mn | NFPP-Ni |
| --- | --- | --- | --- |
| Radiation | Power X-ray (Cu Kα) λ=1.5418 | | |
| Crystal system | Orthorhombic | | |
| Space group | Pn21a (No. 33) | | |
| Lattice parameter *a* (Å) | 17.95190 | 18.01280 | 18.02190 |
| Lattice parameter *b* (Å) | 6.52850 | 6.53330 | 6.53750 |
| Lattice parameter *c* (Å) | 10.65850 | 10.65930 | 10.66330 |
| Unit-cell volume (Å^3^) | 1249.165335 | 1254.418664 | 1256.330448 |
| R_WP_ | 5.04% | 6.51% | 6.44% |

**Table S3.** Optimized atomic fractional positions of NFPP.

| Atom | x | y | z | Occ factor |
| --- | --- | --- | --- | --- |
| Fe1 | 0.3408 | 0.108 | 0.4881 | 0.84 |
| Fe2 | 0.1407 | 0.611 | 0.4911 | 0.84 |
| Fe3 | 0.2506 | 0.329 | 0.7416 | 0.84 |
| P1 | 0.305 | 0.591 | 0.503 | 1 |
| P2 | 0.16 | 0.07 | 0.491 | 1 |
| P3 | 0.5403 | 0.497 | 0.734 | 1 |
| P4 | 0.4361 | 0.146 | 0.7123 | 1 |
| Na1 | 0.4892 | 0.783 | 1.008 | 1 |
| Na2 | 0.2696 | 0.809 | 0.733 | 1 |
| Na3 | 0.3995 | 0.472 | 0.246 | 1 |
| Na4 | 0.4632 | 0.68 | 0.529 | 1 |
| O1 | 0.2299 | 0.542 | 0.625 | 1 |
| O2 | 0.328 | 0.375 | 0.469 | 1 |
| O3 | 0.3404 | 0.794 | 0.508 | 1 |
| O4 | 0.231 | 0.625 | 0.404 | 1 |
| O5 | 0.234 | 0.099 | 0.57 | 1 |
| O6 | 0.1377 | 0.844 | 0.523 | 1 |
| O7 | 0.2245 | 0.066 | 0.392 | 1 |
| O8 | 0.132 | 0.293 | 0.438 | 1 |
| O9 | 0.467 | 0.352 | 0.695 | 1 |
| O10 | 0.5399 | 0.583 | 0.903 | 1 |
| O11 | 0.6158 | 0.319 | 0.739 | 1 |
| O12 | 0.5984 | 0.62 | 0.625 | 1 |
| O13 | 0.4347 | 0.099 | 0.871 | 1 |
| O14 | 0.357 | 0.237 | 0.703 | 1 |
| O15 | 0.4986 | 0.992 | 0.644 | 1 |

**Table S4.** Optimized atomic fractional positions of NFPP-Ni.

| Atom | x | y | z | Occ factor |
| --- | --- | --- | --- | --- |
| Fe1 | 0.3462 | 0.075 | 0.459 | 0.459 |
| Fe2 | 0.141 | 0.607 | 0.4927 | 0.4927 |
| Fe3 | 0.2508 | 0.334 | 0.7454 | 0.7454 |
| P1 | 0.2937 | 0.599 | 0.5064 | 0.5064 |
| P2 | 0.1647 | 0.087 | 0.4997 | 0.4997 |
| P3 | 0.5533 | 0.485 | 0.752 | 0.752 |
| P4 | 0.4497 | 0.144 | 0.7476 | 0.7476 |
| Na1 | 0.4965 | 0.734 | 1.008 | 1.008 |
| Na2 | 0.2827 | 0.796 | 0.706 | 0.706 |
| Na3 | 0.399 | 0.477 | 0.241 | 0.241 |
| Na4 | 0.4812 | 0.674 | 0.514 | 0.514 |
| O1 | 0.2454 | 0.556 | 0.613 | 0.613 |
| O2 | 0.322 | 0.399 | 0.434 | 0.434 |
| O3 | 0.3593 | 0.797 | 0.551 | 0.551 |
| O4 | 0.2074 | 0.607 | 0.474 | 0.474 |
| O5 | 0.2632 | 0.122 | 0.67 | 0.67 |
| O6 | 0.154 | 0.941 | 0.488 | 0.488 |
| O7 | 0.2478 | 0.096 | 0.492 | 0.492 |
| O8 | 0.1119 | 0.272 | 0.47 | 0.47 |
| O9 | 0.4474 | 0.337 | 0.683 | 0.683 |
| O10 | 0.529 | 0.589 | 0.877 | 0.877 |
| O11 | 0.6169 | 0.343 | 0.731 | 0.731 |
| O12 | 0.5877 | 0.588 | 0.613 | 0.613 |
| O13 | 0.4235 | 0.115 | 0.957 | 0.957 |
| O14 | 0.3882 | 0.149 | 0.682 | 0.682 |
| O15 | 0.5003 | 0.975 | 0.656 | 0.656 |
| Ni1 | 0.35 | 0.104 | 0.55 | 0.55 |
| Ni2 | 0.094 | 0.601 | 0.58 | 0.58 |
| Ni3 | 0.3163 | 0.342 | 0.716 | 0.716 |

**Table S5.** Optimized atomic fractional positions of NFPP-Mn.

| Atom | x | y | z | Occ factor |
| --- | --- | --- | --- | --- |
| Fe1 | 0.3377 | 0.096 | 0.499 | 0.837 |
| Mn1 | 0.53 | 0.106 | 0.54 | 0.033 |
| Fe2 | 0.1401 | 0.583 | 0.498 | 0.863 |
| Mn2 | 0.13 | 0.59 | 0.55 | 0.033 |
| Fe3 | 0.2517 | 0.325 | 0.7433 | 0.837 |
| Mn3 | 0.41 | 0.327 | 0.77 | 0.033 |
| P1 | 0.2981 | 0.573 | 0.494 | 1 |
| P2 | 0.1679 | 0.082 | 0.478 | 1 |
| P3 | 0.5592 | 0.485 | 0.75 | 1 |
| P4 | 0.4491 | 0.149 | 0.721 | 1 |
| Na1 | 0.493 | 0.8 | 0.994 | 1 |
| Na2 | 0.2795 | 0.831 | 0.731 | 1 |
| Na3 | 0.3898 | 0.428 | 0.262 | 1 |
| Na4 | 0.4672 | 0.665 | 0.533 | 1 |
| O1 | 0.232 | 0.537 | 0.603 | 1 |
| O2 | 0.331 | 0.417 | 0.464 | 1 |
| O3 | 0.347 | 0.802 | 0.511 | 1 |
| O4 | 0.233 | 0.625 | 0.393 | 1 |
| O5 | 0.244 | 0.094 | 0.58 | 1 |
| O6 | 0.134 | 0.896 | 0.512 | 1 |
| O7 | 0.247 | 0.069 | 0.342 | 1 |
| O8 | 0.135 | 0.302 | 0.444 | 1 |
| O9 | 0.47 | 0.385 | 0.686 | 1 |
| O10 | 0.534 | 0.551 | 0.877 | 1 |
| O11 | 0.618 | 0.3 | 0.748 | 1 |
| O12 | 0.6 | 0.62 | 0.606 | 1 |
| O13 | 0.438 | 0.086 | 0.877 | 1 |
| O14 | 0.372 | 0.217 | 0.697 | 1 |
| O15 | 0.499 | 1.008 | 0.652 | 1 |

**Table S6.** EXAFS data fitting results of NFPP, NFPP-Ni and NFPP-Mn samples.^[17-19]^

| Sample | Path | CN ^a^ | R(Å) ^b^ | σ^2^ (Å^2^)^c^ | ΔE_0_ (eV)^d^ | R factor |
| --- | --- | --- | --- | --- | --- | --- |
| Fe K-edge (Ѕ02=0.841) | | | | | | |
| Fe foil | Fe-Fe | 8* | 2.459±0.009 | 0.0035 | 4.5 | 0.0024 |
|  | Fe-Fe | 6* | 2.837±0.010 | 0.0032 |  |  |
| NFPP | Fe-O | 5.8±0.3 | 2.055±0.008 | 0.0143 | -0.2 | 0.0033 |
|  | Fe-O-Fe | 2.9±0.4 | 3.059±0.015 | 0.0113 | -5.4 |  |
| NFPP-Mn | Fe-O | 6.0±0.2 | 2.092±0.022 | 0.0135 | -1.0 | 0.0046 |
|  | Fe-O-Fe/Ni | 5.0±0.4 | 3.106±0.029 | 0.0181 | -2.2 |  |
| NFPP-Ni | Fe-O | 6.1±0.2 | 2.070±0.008 | 0.0138 | -0.8 | 0.0053 |
|  | Fe-O-Fe/Mn | 5.4±0.5 | 3.092±0.021 | 0.0199 | -2.6 |  |
| Ni K-edge (Ѕ02=0.766) | | | | | | |
| Ni foil | Ni-Ni | 128 | 2.483±0.002 | 0.0059 | -0.6 | 0.0016 |
| NFPP-Ni | Ni-O | 5.9±0.2 | 2.048±0.004 | 0.0089 | -4.2 | 0.0025 |
|  | Ni-O-Ni | 4.2±0.3 | 3.097±0.014 | 0.0140 | 0.5 |  |

*^a^CN*, coordination number; *^b^R*, the distance between absorber and backscatter atoms; *^c^σ*^2^, the Debye Waller factor value; *^d^ΔE*_0_, inner potential correction to account for the difference in the inner potential between the sample and the reference compound; *R* factor indicates the goodness of the fit. *S*0^2^ was fixed to 0.841 and 0.766, according to the experimental EXAFS fit of Fe foil and Ni foil by fixing *CN* as the known crystallographic value. * This value was fixed during EXAFS fitting, based on the known structure of Fe and Ni. Fitting conditions: *k* range: 2.0-10.0; *R* range: 1.0-3.0; fitting space: R space; *k*-weight = 3. A reasonable range of EXAFS fitting parameters: 0.700 < *Ѕ*_0_^2^ < 1.000; *CN >* 0; *σ*^2^ > 0 Å^2^; |Δ*E*_0_| < 15 eV; *R* factor < 0.02.

**Table S7.** The average resistivity of NFPP, NFPP-Ni and NFPP-Mn at different pressure.

| Pressure (Mpa) | Average resistivity | | |
| --- | --- | --- | --- |
|  | NFPP (KΩ-cm) | NFPP-Ni (MΩ-cm) | NFPP-Mn (MΩ-cm) |
| 2 | 9147.06 | 113.69 | 251.55 |
| 4 | 6298.97 | 93.54 | 183.52 |
| 6 | 6228.26 | 81.13 | 145.98 |
| 8 | 6123.60 | 71.14 | 121.43 |
| 10 | 6206.90 | 64.81 | 111.71 |
| 12 | 6129.41 | 60.39 | 97.90 |
| 14 | 5833.33 | 56.00 | 86.25 |
| 16 | 5609.76 | 52.80 | 78.59 |
| 18 | 5209.88 | 50.55 | 71.56 |
| 20 | 4556.96 | 47.95 | 66.80 |
| 22 | 3948.72 | 46.39 | 62.03 |
| 24 | 3194.81 | 45.07 | 57.95 |
| 26 | 2519.48 | 42.96 | 54.58 |
| 28 | 2052.63 | 41.41 | 49.58 |
| 30 | 1689.19 | 40.43 | 45.43 |

**Table S8.** Simulation results of the EIS spectra using equivalent circuit shown in Figure S31.

| Sample | NFPP | NFPP-Ni | NFPP-Mn |
| --- | --- | --- | --- |
| R_s_ (Ω) | 3.33 | 2.433 | 3.478 |
| R_ct_ (Ω) | 292.1 | 259.8 | 310.4 |

**Table S9.** Computing results of DOS.

|  | NFPP | NFPP-Zn | NFPP-Ni | NFPP-Mn |
| --- | --- | --- | --- | --- |
| Fe d center | -1.058 | -1.116 | -1.003 | -0.559 |
| O p center | -3.715 | -3.196 | -3.618 | -3.600 |
| Fe d – O p | 2.657 | 2.080 | 2.615 | 3.041 |
| Fermi | 0 | 0 | 0 | 0 |

**Table S10.** The Fe–O covalence degree of NFPP, NFPP-Zn, NFPP-Ni and NFPP-Mn.

|  | NFPP | NFPP-Zn | NFPP-Ni | NFPP-Mn |
| --- | --- | --- | --- | --- |
| Fe6 – O14 covalence degree | 0.18 | 0.19 | 0.19 | 0.13 |
| Fe3 – O19 covalence degree | 0.18 | 0.21 | 0.19 | 0.16 |
| Fe2 – O26 covalence degree | 0.12 | 0.16 | 0.13 | 0.11 |
| Fe7 – O3 covalence degree | 0.19 | 0.21 | 0.19 | 0.16 |
| Aerage value | 0.16 | 0.19 | 0.17 | 0.14 |

**Reference**

1. G. Kresse, J. Hafner. Ab initio molecular dynamics for open-shell transition metals. Phys. Rev. **48**, 13115 (1993). https://doi.org/10.1103/PhysRevB.48.13115
2. G. Kresse, J. Furthmüller. Software VASP, vienna (1999). Phys. Rev. **54**, 169 (1996).
3. John P. Perdew, K. Burke, M. Ernzerhof, Phys. Rev. Lett. **77**, 3868 (1996).
4. W. Kohn, L. J. Sham, Self-consistent equations including exchange and correlation effects. Phys. Rev. **140**, A1133 (1965). https://doi.org/10.1103/PhysRev.140.A1133
5. V. Wang, N. Xu, J.-C. Liu, G. Tang, W.-T. Geng, VASPKIT: a user-friendly interface facilitating high-throughput computing and analysis using VASP code. Comput. Phys. Commun. **267**, 108033 (2021). <https://doi.org/10.1016/j.cpc.2021.108033>
6. K. Momma, F. Izumi, VESTA: a three-dimensional visualization system for electronic and structural analysis. J. Appl. Crystallogr. **41**, 653 (2008). https://doi.org/[10.1107/S0021889808012016](https://doi.org/10.1107/S0021889808012016)
7. X. Wang, H. Li, W. Zhang, X. Ge, L. He et al., Unlocking fast and highly reversible sodium storage in Fe-based mixed polyanion cathodes for low-cost and high-performance sodium-ion batteries. J. Mater. Chem. A. **11**, 6978 (2023). https://doi.org/10.1039/d3ta00014a
8. L. Huang, C. Liu, L. Bao, Y. Chen, Y. Jiang et al., Large scalable preparation of Ti-doped Na_4_Fe_3_(PO_4_)_2_P_2_O_7_ as cathode material for high rate and long-life sodium-ion batteries. ACS Appl. Energy Mater. **6**, 11541 (2023). https://doi.org/10.1021/acsaem.3c01910
9. X. Wu, Y. Cui, Y. Yao, Y. Gao, C. Guo et al., Effect of K^+^-doping on oxygen vacancies reduction, kinetics and cycling stability of Na_4_Fe_3_(PO_4_)_2_(P_2_O_7_)/C cathodes for sodium-ion batteries. J. Energy Storage **132**, 117661 (2025). https://doi.org/10.1016/j.est.2025.117661
10. X. Wang, S. Li, Z. Fang, G. Zhang, C. Han et al., Air-stable and robust iron-based phosphate cathodes for fast-charged and wide-temperature range sodium ion storage. Nano Energy **144**, 111397 (2025). https://doi.org/10.1016/j.nanoen.2025.111397
11. Y. Subaşı, G. Ek, P. Törnblom, M. Hirsbrunner, E. Johannesson et al., Mn/Ni-doped Na_4_Fe_3_(PO_4_)_2_(P_2_O_7_) cathodes: structural, electrochemical, and spectroscopic insights for sodium-ion batteries. J. Power Sources **663**, 238901 (2025). https://doi.org/10.1016/j.jpowsour.2025.238901
12. W. Fei, Y. Sui, Y. Wang, K. Sun, X. Zhang et al., Regulating Na/Mn antisite defects and reactivating anomalous jahn−teller behavior for Na_4_Fe_1.5_Mn_1.5_(PO_4_)_2_(P_2_O_7_) cathode material with superior performance. ACS Nano **19**, 8303 (2025). https://doi.org/10.1021/acsnano.4c18614
13. Y. Xin, Q. Wang, Y. Wang, M. Wang, F. Wu et al., Experimental and theoretical investigation of cobalt and manganese substitution in Na_4_Fe_3_(PO_4_)_2_P_2_O_7_ as a high energy density cathode material for sodium-ion batteries. Chem. Eng. J. **483**, 149438 (2024). https://doi.org/10.1016/j.cej2024.149438
14. Y. Cao, C. Yang, Y. Liu, X. Xia, D. Zhao et al., A new polyanion Na_3_Fe_2_(PO_4_)P_2_O_7_ cathode with high electrochemical performance for sodium-ion batteries. ACS Energy Lett. **5**, 3788 (2020). https://doi.org/10.1021/acsenergylett.0c01902
15. N. Wang, J. Ma, Z. Liu, J. Xu, D. Zhao et al., An air-stable iron/manganese-based phosphate cathode for high performance sodium-ion batteries. Chem. Eng. J. **433**, 133798 (2022). https://doi.org/10.1016/j.cej.2021.133798
16. H. Funke, A. C. Scheinost, M. Chukalina. Wavelet analysis of extended x-ray absorption fine structure data. Phys. Rev. B. **71**, 094110 (2005). https://doi.org/10.1103/PhysRevB.71.094110
17. B. Ravel, M. Newville, J. Synchrot. Radiat. ATHENA, ARTEMIS, HEPHAESTUS: data analysis for X-ray absorption spectroscopy using IFEFFIT. **12**, 537 (2005). https://doi.org/10.1107/S0909049505012719
18. S. I. Zabinsky, J. J. Rehr, A. Ankudinov, R. C. Albers, M. J. Eller. Multiple-scattering calculations of X-ray-absorption spectra. Phys. Rev. B. **52**, 2995 (1995). https://doi.org/10.1103/PhysRevB.52.2995
